# Supplementary material for: Inherited defects of piRNA biogenesis cause transposon de-repression, impaired spermatogenesis, and human male infertility
Source: Nat Commun. 2024 Aug 9;15:6637. doi: 10.1038/s41467-024-50930-9 (PMC11316121; doi:10.1038/s41467-024-50930-9)
Supplement: Supplementary file 1 — Supplementary Information [file 41467_2024_50930_MOESM1_ESM.pdf]

**Supplementary Information**

# Inherited defects of piRNA biogenesis cause transposon de-repression, impaired spermatogenesis, and human male infertility

**Supplementary Tables**

Supplementary Table 1. Human piRNA biogenesis genes included in variant screening.

Supplementary Table 2. Clinical data of infertile men with high impact bi-allelic variants in genes of the piRNA pathway.

Supplementary Table 3. Primer information.

Supplementary Table 4. Antibody information.

**Supplementary Figures**

Supplementary Figure 1. Schematic overview on piRNA biogenesis in the mammalian testi

Supplementary Figure 2. Pie chart illustrating two-tiered biological sub-processes clustering to identified main biological processes identified in gene ontology (GO) analysis.

Supplementary Figure 3. Testicular phenotypes of patients with biallelic high impact variants in genes of the piRNA pathway.

Supplementary Figure 4. Genetic data on homozygous high impact variants in *PIWIL1*, *PIWIL2* and *GTSF1*.

Supplementary Figure 5. Genetic data on homozygous high impact variants in *MOV10L1* and *PLD6*.

Supplementary Figure 6. Genetic data on biallelic high impact variants identified in *GPAT2*.

Supplementary Figure 7. Genetic data on biallelic high impact variants identified in *GPAT2*, *PNLDC1* and *HENMT1*.

Supplementary Figure 8. Genetic data on biallelic high impact variants identified in *DDX4* and *MAEL*.

Supplementary Figure 9. Testicular expression profile of piRNA pathway components DDX4 and TDRD1 in control and identified variant carriers.

Supplementary Figure 10. Genetic data on biallelic high impact variants identified in *TDRD1* and *TDRD9*.

Supplementary Figure 11. Genetic data on biallelic high impact variants identified in *TDRD12*.

Supplementary Figure 12. Expression of key piRNA pathway components in testicular tissue of variant carriers.

Supplementary Figure 13. Testicular expression profile of piRNA pathway components HENMT1 and MAEL in control and variant carriers.

Supplementary Figure 14. Testicular expression profile of piRNA pathway components PIWIL1 and GTSF1 in controls and variant carriers.

Supplementary Figure 15. Testicular expression profile of piRNA pathway components PLD6 and GPAT2 in control and variant carriers.

Supplementary Figure 16. Impact of biallelic variants in piRNA biogenesis genes on amount of pachytene piRNA and transposon expression.

**Supplementary References**

## Supplementary Tables

### Supplementary Table 1. Human piRNA biogenesis genes included in variant screening

| Mouse | Human | Cellular localisation | Function | Testicular phenotype in knockout mice | Ref. |
|---|---|---|---|---|---|
| *Miwi* | *PIWIL1* | cytoplasmic granules | RNA slicing | RsA | [1] |
| *Mili* | *PIWIL2* | cytoplasmic granules | RNA slicing | MeiA (zygotene) | [2] |
| - | *PIWIL3* | unkown | unknown | n.a. | - |
| *Miwi2* | *PIWIL4* | cytoplasmic granules and nucleus | de novo methylation | MeiA (zygotene) | [3] |
| *Gtsf1* | *GTSF1* | cytoplasmic granules | enhancer of PIWI slice activity | MeiA (zygotene)/ apoptotic germ cells | [4] |
| *Pld6* | *PLD6* | mitochondrial outer membrane | endonuclease | MeiA (zygotene)/ apoptotic spermatocytes | [5] |
| *Gpat2* | *GPAT2* | mitochondrial outer membrane | Glycerol-3-phosphate O-acyltransferase/ | MeiA (zygotene) apoptotic spermatocytes and neonatal spermatogonia | [6] |
| *Mov10l1* | *MOV10L1* | cytoplasmic granules | RNA helicase | MeiA (zygotene) | [7] |
| *Gasz* | *ASZ1* | cytoplasmic granules | stabilizing of PIWIL2 | MeiA (zygotene) | [8] |
| *Pnldc1* | *PNLDC1* | n.a. | trimming of piRNA precursor | MeiA (pachytene)/ ES+ | [9–11] |
| *Henmt1* | *HENMT1* | cytoplasmic granules | RNA methyltransferase | reduced amount of elongated spermatids, (head and tail defects) | [12] |
| *Fkbp6* | *FKBP6* | cytoplasm | chaperone protein | MeiA (pachytene) | [13] |
| *Mael* | *MAEL* | cytoplasmic granules/nucleus | Nucleus-cytoplasm shuttling protein | MeiA (pachytene)/RsA | [14] |
| *Mvh* | *DDX4* | cytoplasmic granules | RNA helicase | MeiA (zygotene) | [15,16] |
| *Tdrd1* | *TDRD1* | cytoplasmic granules | scaffold protein | MeiA (pachytene)/ RsA | [17] |
| *Tdrkh* | *TDRKH* | cytoplasmic granules | scaffold protein | MeiA (zygotene) | [18] |
| *Rnf17* | *RNF17* | cytoplasmic granules | scaffold protein | RsA | [19] |
| *Tdrd5* | *TDRD5* | cytoplasmic granules/nucleus | scaffold protein | MeiA (zygotene)/RsA | [20,21] |
| *Tdrd9* | *TDRD9* | cytoplasmic granules | scaffold protein | MeiA | [22] |
| *Tdrd12* | *TDRD12* | cytoplasmic granules | scaffold protein | MeiA (zygotene) | [23] |
| *Mybl1* | *MYBL1* | nucleus | transcription factor | MeiA/ apoptotic spermatocytes | [24] |
| *Uhrf1* | *UHRF1* | cytoplasm/nucleus | unknown | MeiA (pachytene) | [25] |
| *Hsp90aa1* | *HSP90AA1* | cytoplasm | chaperone protein | MeiA | [26] |
| *Btbd18* | *BTBD18* | nucleus | transcription factor | RsA | [27] |

**Supplementary Table 2: Clinical data of infertile men with high impact bi-allelic variants in genes of the piRNA pathway.**

| **Individual/ gene variant** | **Age, origin** | **Fertility parameters** (FSH/LH/T/TVr;l) | **Semen analysis** | **Testicular phenotype** (r/l in % of all tubuli) | **TESE outcome/ additional clinical features** |
|---|---|---|---|---|---|
| **M928;***DDX4*<br>c.[1532C>T];[1532C>T] | 36 y,<br>Libya | 2.7/2.1/19.3<br>22;15 | **Crypto** | **RsA** (0/0 ES, 5/6 RS, 93/87 SPC, 2/5 SPG, 0/0 SCO, 0/1 TS) | negative |
| **M2546*;***FKBP6*:<br>c.[508_529dup];[832C>T] | 28 y,<br>Kyrgyzstan | 3.9/4.5/18.1<br>**10**;12 | **Crypto** | **RsA** (0/0 ES; 5/33 RS, 90/64 SPC, 4/3 SPG, 1/0 SCO, 0/1 TS) | negative |
| **M2548*;***FKBP6*<br>c.[508_529dup];[508_529dup] | 26 y,<br>Germany | 1.8/4.0/18.6<br>39;23 | **Crypto** | **RsA** (0/0 ES, 42/29 RS, 57/67 SPC, 2/2 SPG, 0/1 SCO, 0/1 TS) | negative |
| **M1400*;***FKBP6*<br>c.[589-2A>G];[589-2A>G] | 43 y,<br>Syria | **7.7**/3.5/13.4<br>**7**;14 | **Crypto** | **RsA** (0/0 ES, 27/13 RS, 39/45 SPC, 5/3 SPG, 5/6 SCO, 25/34 TS) | negative |
| **MI-0042P;***GPAT2:*<br>c.[146G>A];[146G>A] | 31 y, Irish | 5.8/n.a./**11.3**<br>25;25 | **ExtOligo** | n.d. | negative |
| **M2556;***GPAT2*:<br>c.[1156-1G>A];[1156-1G>A] | 21 y,<br>Germany | **17.4/15.6**/23.8<br>22;16 | **Crypto** | **MeiA** (0/0 ES, 0/0 RS, 38/56 SPC, 41/7 SPG, 22/32 SCO, 0/6 TS) | negative |
| **M13;***GPAT2*:<br>c.[1130A>G ];[1954C>T] | 29 y,<br>Germany | **8.9/**4.2**/**16.1<br>**9**;**9** | **Azoo** | **SCO** (0/0 ES, 0/0 RS, 0/0 SPC, 0/0 SPG, 100/100 SCO, 0/0 TS) | negative |
| **M454;***GPAT2*:<br>c.[1130A>G];[146G>A] | 23 y,<br>Germany | **9,5**/5.6/**11.1**<br>15;19 | **Azoo** | **SCO** (0/0 ES, 0/0 RS, 0/0 SPC, 0/0 SPG, 100/100 SCO, 0/0 TS) | negative |
| **17-051;***GPAT2*:<br>c.[1388C>T];[1388C>T] | 31 y,<br>Morocco | **14,7**/5.6/19.4<br>12;15 | **Azoo** | **SCO** (0/0 ES, 0/0 RS, 0/0 SPC, 0/0 SPG, 100/100 SCO, 0/0 TS) | negative |
| **15-0730;***GPAT2*:<br>c.[1388C>T];[1388C>T] | 32 y,<br>Morocco | **12,5**/4.7/13<br>20;20 | **Azoo** | **SCO** (0/0 ES, 0/0 RS, 0/0 SPC, 0/0 SPG, 100/100 SCO, 0/0 TS) | negative |
| **M690;***GPAT2*:<br>c.[1879C>T];[1879C>T] | 32 y,<br>Germany | **21.2/**8.1**/11.8**<br>**9**;13 | **Azoo** | **MeiA** (0/0 ES, 0/0 RS, 7/5 SPC, 24/6 SPG, 16/39 SCO, 52/50 TS) | negative |
| **M1844;***GPAT2*:<br>c.[1879C>T];[1879C>T] | 28 y,<br>Turkey | **15.8/**3.7**/7.2**<br>**5**;**5** | **Azoo** | **SCO**<br>(no further specification available) | negative |
| **M2043;***GTSF1*:<br>c.[97C>A];[97C>A] | unknown | n.a. | **Azoo** | **MeiA**<br>(no further specification available) | negative |
| **M2243;***GTSF1*:<br>c.[221_222del];[221_222del] | 32 y,<br>Turkmenistan | 6.1/5.6/12.1<br>**7**;**6** | **Azoo** | **MeiA** (0/0 ES, 0/0 RS, 20/13 SPC, 71/78 SPG, 9/6 SCO, 0/3 TS) | negative |
| **M3079;***HENMT1*:<br>c.[400A>T];[400A>T] | 26 y,<br>unknown | **7.1**/5.9/**4.7**<br>15;15 | **Azoo** | **RsA**<br>(no further specification available) | n.d./chromosomal aberration† |
| **M2435;***MAEL*:<br>c.[799C>T];[908+1G>C] | 54 y,<br>Pakistan | **10.6/**4.3**/**12.3<br>**11**;**10** | **Azoo** | **MeiA** (0/0 ES, 0/0 RS, 25/54 SPC, 20/26 SPG, 16/14 SCO, 39/6 TS) | negative |
| **TP17;***MOV10L1*:<br>c.[2179+3A>G];[2179+3A>G] | 33 y,<br>Turkey | 6.1/4.4**/7.8**<br>**4;4** | **Azoo** | **Spermatogonia arrest** | negative |
| **M1948;***MOV10L1*:<br>c.[2258T>C];[2258T>C] | 32 y,<br>Iraq | **17.9/**3.4**/**16.9<br>12;14 | **Azoo** | n.d. | n.d. |
| **TP24;***MOV10L1*:<br>c.[3115G>A];[3115G>A] | 31 y,<br>Turkey | **14.7/**8.3**/**18.8<br>**6;5** | **Azoo** | **SCO** | negative |
| **MIProband02199;***MOV10L1*:<br>c.[3268G>T];[3268G>T] | 28 y,<br>Turkey | **14**/n.a./n.a.<br>18;15 | **Azoo** | **SCO** | negative |
| **M2006;***PIWIL1*:<br>c.[688C>T];[688C>T] | 38 y,<br>Pakistan | **7.4**/4.8/**9.1**<br>16;**11** | **Azoo** | **RsA** (0/0 ES, 6/0 RS, 91/15 SPC, 3/2 SPG, 0/7 SCO, 1/75 TS) | negative |

| | | | | | |
|---|---|---|---|---|---|
| **TP32;***PIWIL2*:<br>c.[839A>C];[839A>C] | 24 y,<br>Turkey | **8.4**/4.4/18.5<br>**5;6** | **Azoo** | **SCO** | negative |
| **M2949;***PIWIL2*:<br>c.[1697G>A];[1697G>A] | 37 y,<br>Syria | 4.7/5.3/**9.9**<br>13;13 | **Azoo** | n.d. | n.d. |
| **M2173;***PLD6*:<br>c.[1A>T];[1A>T] | 28 y,<br>unknown | **8.3/**2.9/**6.0**<br>12;18 | **Azoo** | **SCO**<br>(no further specifications available) | |
| **M2803;***PLD6*:<br>c.[469del];[469del] | 32 y,<br>Germany | **34.3/**4.2/13.3<br>16;19 | **Azoo** | **SCO** (0/0 ES, 0/0 RS, 0/0 SPC,<br>0/0 SPG, 96/92 SCO, 4/8 TS) | negative/<br>kidney carcinoma |
| **M3274;***PNLDC1*:<br>c.[790G>T];[790G>T] | 33 y,<br>Turkey | 2.0/**1.1**/**3.9**<br>30;28 | **Crypto** | n.d. | n.d. |
| **M1125;***PNLDC1*:<br>c.[1058A>G];[1058A>G] | 25 y,<br>Romania | 1.7/2.3/**10.7**<br>16;15 | **Crypto** | **Es+** (1/4 ES, 60/51 RS, 33/43 SPC,<br>5/1 SPG, 2/1 SCO, 1/0 TS) | negative |
| **M1648;***TDRD1*:<br>c.[887C>A];[887C>A] | 31 y,<br>Afghanistan | **27.7/**7.9**/**15.1<br>**4;4** | **Azoo** | **MeiA** (0/0 ES, 0/0 RS, 39/14 SPC,<br>41/44 SPG, 9/22 SCO, 10/20 TS) | negative |
| **M2842;***TDRD9*:<br>c.[1243G>T];[1243G>T] | 36 y,<br>unknown | 4.0/2.4/16.6.<br>24;21 | **ExtOligo**<br>0% motile | n.d. | n.d./chromosomal<br>aberration# |
| **M800;***TDRD9*:<br>c.[3148dup];[3148dup] | 42 y,<br>Germany | 3.1/**1.8**/**7.8**<br>25;20 | **ExtOligo**<br>0% motile | **ES+** (15/11 ES, 17/53 RS, 17/3 SPC,<br>9/2 SPG, 1/0 SCO, 50/3 TS) | positive |
| **M3007**;*TDRD9*:<br>c.[3716+3A>G];[3716+3A>G] | 37 y,<br>unknown | 3.4/4.3/17.0<br>14;**7** | **ExtOligo**<br>5% motile | n.d. | n.d. |
| **M2442**;*TDRD9*:<br>c.[3826G>T];[3826G>T] | 33 y,<br>Turkey | **31/13.9/**24.1<br>**10;10** | **Crypto** | n.d. | negative |
| **M2662**;*TDRD12:*<br>c.[287A>C];[287A>C] | 34 y,<br>Egypt | **34.4**/9.8/23.3<br>16;14 | **Azoo** | **SCO** (0/0 ES, 0/0 RS, 0/0 SPC, 0/0<br>SPG, 75/55 SCO, 25/45 TS) | negative |
| **M1642**‡;*TDRD12:*<br>c.[593A>G];[593A>G] | 26 y,<br>Morocco | **11.2**/3.1/25.5<br>**7.3**;**6.6** | **Azoo** | **SCO** (0/0 ES, 0/0 RS, 0/0 SPC, 0/0<br>SPG, 100/98 SCO, 0/2 TS) | negative |
| **TP5;***TDRD12:*<br>c.[963+1G>T];[963+1G>T] | 50 y,<br>Turkey | n.a./n.a./n.a.<br>**6;9** | **Azoo** | **MeiA** (few spermatocytes) | negative |
| **M2227**;*TDRD12:*<br>c.[986G>A];[986G>A] | 29 y,<br>Kazakhstan | **8.5**/4.1/20.2<br>19;20 | **Azoo** | **RsA** (0/0 ES; 31/31 RS, 66/66 SPC,<br>2/3 SPG, 0/0 SCO, 0/1 TS) | negative |
| **M2940**;*TDRD12:*<br>c.[2419C>T];[2419C>T] | 36 y,<br>Syria | 3.7/**1.3**/**10.6**<br>**7**;7 | **Crypto** | n.d. | n.d. |
| **M2317**;*TDRD12:*<br>c.[2432G>A];[2432G>A] | 36 y,<br>Syria | **8.6**/4.9/23<br>12;14 | **Azoo** | **ES+** (9/6 ES, 17/30 RS, 73/61 SPC,<br>1/1 SPG, 0/1 SCO, 0/0 TS) | negative |
| **M2595**;*TDRD12:*<br>c.[3157del];[3157del] | 43 y,<br>Iraq | **31.5**/7.3/**9.2**<br>**7;6** | **Azoo** | **ES+** (0/3 ES, 0/3 RS, 36/42 SPC,<br>17/6 SPG, 16/14 SCO, 31/33 TS) | negative |

FSH: follicle stimulating hormone (IU/L), LH: luteinizing hormone (IU/L), NA: not available/applicable, T: testosterone (nmol/L), TV: testicular volume right/left (mL), y: years. Reference values: FSH 1-7 IU/L, LH 2-10 IU/L, T >12 nmol/L, TV >12 mL per testis. Values outside of the reference range are shown in bold. ES+: elongated spermatids present; MeiA: meiotic arrest; SCO: Sertoli cell-only; RsA: round spermatid arrest; ExtOligo: extreme oligozoospermia; ES: elongated spermatids; RS: round spermatids; SPC: spermatocytes; SPG: spermatogonia; TS: tubular shadows; n.d.: not done; *already described[28]; ‡already described[29]; †46,XY,der(15;21)(q10;q10);#45,XY,der(13;14)(q10;q10)[26]/ 46,XY[4].

**Supplementary Table 3. Primer information.**

| Method | Target | Primer sequences (5´-3´) |
|---|---|---|
| Sanger sequencing | *DDX4* *(*NM_024415) c.1532C>T | Forw:TGGATAGCAAATCCACTTGTAATTACC Rev:TCAAACTTAATGGTGGAGAAACGA |
| Long range PCR | *GPAT2* | Forw:TTCTGACCCTGAGTTGTCCC Rev:GTCAGCCTCTCCGAAGTCAT |
| Sanger sequencing | *GPAT2* (NM_001321526) c.1156-1G>A | Forw:GCCATACCCCACACTCAGAC Rev:GCCCTTTTCCCTGCAGGTAT |
| Sanger sequencing | *GPAT2* (NM_001321526) c.1879C>T | Forw:AGAGGCACACATGTCCAGTGC Rev:GCATATTGCTGCTGAGCCAG |
| Sanger sequencing | *GPAT2* (NM_001321526) c.1954C>T | Forw:GCATATTGCTGCTGAGCCAG Rev:GTCAGCCTCTCCGAAGTCAT |
| Sanger sequencing | *GPAT2* (NM_001321526) c.1130A>G | Forw:TAGCTGCTCCAGGGTCTGTC Rev:ATCATCTGGCAGCACCTGTC |
| Sanger sequencing | *GPAT2* (NM_001321526) c.146G>A | Forw:CACCCTACCACCACTTAGGC Rev:AGAAGGAGAGTGCCAGAGGT |
| Sanger sequencing | *GPAT2* (NM_001321526) c.1388C>T | Forw:GCCCAGAGAAGCCTACATCA Rev:AGAGGCTGTGGGTTGATGTG |
| Sanger sequencing | *GTSF1* (NM_144594) c.97C>A | Forw:GCAGGATTTCTCATTGTTAGCCA Rev:CCCTGAGATTTCTGGTTCTGCT |
| Sanger sequencing | *GTSF1* (NM_144594) c.221_222del | Forw:ACCAGGCTGTTCAGGAGAGA Rev:TCGTTCCAATACCCAATCCTAAA |
| Sanger sequencing | *HENMT1* (NM_144584) c.400A>T | Forw:ACACCCCCTTTCCACACTTG Rev:TGCCTGGCCCGAATAATTGT |
| Sanger sequencing | *MAEL* (NM_032858.3) c.799C>T | Forw:TCCCAACCCCCACATTTTGA Rev:ACAGTTTATTTGGCAGGTTTGTT |
| Sanger sequencing | *MAEL* (NM_032858.3) c.908+1G>C | Forw:TGTCACTAATGTGCCAGAAGC Rev:ACAGTGTATGAGGGTTCCCT |
| Sanger sequencing | *MOV10L1* (NM_018995) c.2258T>C | Forw:CGTGTCCACAGCTGTTGAGA Rev:CGACACATGAACAGCAGCAC |
| Sanger sequencing | *MOV10L1* (NM_018995) c.2179+3A>G | Forw:CGTGTCCACAGCTGTTGAGA Rev:CGACACATGAACAGCAGCAC |
| Sanger sequencing | *MOV10L1* (NM_018995) c.3115G>A | Forw:GAAATACCAGCCAGCCCTCC Rev:TCTGGCCGTAGAAAAGCTGG |
| Sanger sequencing | *PIWIL1* (NM_004764) c.688C>T | Forw:AACAGCCAGACACAGCGTG Rev:TCAGTTGGGCTGCAATCTTGA |
| Sanger sequencing | *PIWIL2* (NM_018068) c.1697G>A | Forw:ATAAATGTAGGTTTTTATTGACAGGGA Rev:AGTCTATGGTCAGGCAAAGGG |
| Sanger sequencing | *PIWIL2* (NM_018068) c.839A>C | Forw:GCAGGTGTCCTTTGGTGGTA Rev:ACCCCTCAAAACACCAGTCC |

| | | |
|---|---|---|
| Sanger sequencing | *PLD6*<br>(NM_178836)<br>c.469del | Forw:GGCAGGTCGTGACTGAAGTT<br>Rev:TGTCCCCTCTGTGGTTCTCA |
| Sanger sequencing | *PLD6*<br>(NM_178836)<br>c.1A>T | Forw:CTGAGACGGGAAGAACAGCG<br>Rev:ATTGGCAGGACGTGATAGGG |
| Sanger sequencing | *PNLDC1*<br>(NM_001271862)<br>c.1058A>G | Forw:TTTCCCCTTTCCTGTTGCCC<br>Rev:GGTCAAGGACAAGCTCAGCT |
| Sanger sequencing | *TDRD1*<br>(NM_198795)<br>c.887C>A | Forw:CGAATTCAAACACCCAGGGG<br>Rev:GGGAGGAAGCAAGGTGAACA |
| Sanger sequencing | *TDRD12*<br>(NM_001366102)<br>c.3157del | Forw:GGCCCTGTGGGAATGTCTAT<br>Rev: GGTATCTTAGCATCTTCGCGT |
| Sanger sequencing | *TDRD12*<br>(NM_001366102)<br>c.986G>A | Forw:ACTGACGATCTGGAATGCCA<br>Rev:AGACAACATCACTCTTAAGTCTCT |
| Sanger sequencing | *TDRD12*<br>(NM_001366102)<br>c.593A>G | Forw:TGCTACAGTGCGTATGTCCA<br>Rev:TGGCCAGAGTGTAAGTGCTG |
| Sanger sequencing | *TDRD12*<br>(NM_001366102)<br>c.2419C>T | Forw:GACTGGGATAGATGATCCAC<br>Rev:AGCTGTGCACCTTACTGCAG |
| Sanger sequencing | *TDRD12*<br>(NM_001366102)<br>c.963+1G>T | Forw:ACTGACGATCTGGAATGCCA<br>Rev:AGACAACATCACTCTTAAGTCTCT |
| Sanger sequencing | *TDRD12*<br>(NM_001366102)<br>c.3157del | Forw:GGCCCTGTGGGAATGTCTAT<br>Rev:GGTATCTTAGCATCTTCGCGT |
| Sanger sequencing | *TDRD12*<br>(NM_001366102)<br>c.2432G>A | Forw:GACTGGGATAGATGATCCAC<br>Rev:AGCTGTGCACCTTACTGCAG |
| Sanger sequencing | *TDRD9*<br>(NM_153046)<br>c.3826G>T | Forw:GTGGTTTGGGGTGGAATCCA<br>Rev:GGACCAGAGGTGTGTGTCTG |
| Sanger sequencing | *TDRD9*<br>(NM_153046)<br>c.1243G>T | Forw:TGGTAGGGCCTTCTTTGAAAGA<br>Rev:TCCCAGGCACAAGTCTCCC |
| Sanger sequencing | *TDRD9*<br>(NM_153046)<br>c.3716+3A>G | Forw:CTCTTGGCCCTGGGAAAAAT<br>Rev:GAGCCTCATTCCTAGCTCCA |
| Minigene-Assay | *GPAT2*<br>(NM_001321526)<br>c.1156-1G>A | Forw:TGAGATAAAACTGAGCAGGGTGG<br>Rev: AAGGTATCACAGAGCACCTCAG |
| Minigene-Assay | *MOV10L1*<br>(NM_018995)<br>c.2179+3A>G | Forw:GGAAAGCAGGTAGGATGTACC<br>Rev:GGCTGTCAACCGATTCTGCGG |
| Minigene-Assay | *MAEL*<br>(NM_032858.3)<br>c.908+1G>C | Forw:CACCTGAAGTTTTAGTCTTCAAGTAACCTCA<br>Rev:CTTTGTTTTGAAAGAGCATTGTGG |
| Minigene-Assay | *TDRD12*<br>(NM_001366102)<br>c.963+1G>T | Forw:TAATACGACTCACTATAGGGAG<br>Rev:AGGCACAGTCGAGGCTGATCA |
| Minigene-Assay | *TDRD9*<br>(NM_153046)<br>c.3716+3A>G | Forw:CACCGCCATTTTCTGACTGCTTTGG<br>Rev:AGCCTCATTCCTAGCTCCAT |
| Long read Sequencing | *MAEL*<br>(NM_032858.3)<br>c.799C>T/<br>c.908+1G>C | Forw:GGGAACTGGCCACCTATCTA<br>Rev:CCTCACCCCTCTAGTACCCT |

| | | |
|---|---|---|
| Long read Sequencing | *GPAT2* (NM_001321526) c.146G>A/c.1130A>G | Forw:TTCTGACCCTGAGTTGTCCC<br>Rev:GTCAGCCTCTCCGAAGTCAT |
| Cloning | *PLD6* (NM_178836.4) | Forw:GGTGGTAAGCTTAGACTCCGCTGCGGCGGCGTG<br>Rev:ACCACCGAATTCTTAGGTTTGGCTTTCGCTGGA |
| | *PLD6-HA* | Forw:TACCCATACGATGTTCCAGATTACGCTTAAGAATTCTGCAGATATCCAGCACAGTGG<br>Rev: GGTTTGGCTTTCGCTGGAGGTG |
| Mutagenesis | *PLD6* (NM_178836.4) *c.1A>G* | Forw:GATTAGCGGCGGCTTGGGACGGTTGAGTTG<br>Rev:CAACTCAACCGTCCCAAGCCGCCGCTAATC |

## Supplementary Table 4. Antibody information

| Epitope | Application | Source, Species, | Dilution, special protocol requirements |
|---|---|---|---|
| CREM | IHC | Sigma Aldrich, HPA001818, rabbit, polyclonal | 1:2000 in 5% BSA/TBS, no antigen retrieval |
| γH2AX | | Merck, 05-636, mouse, monoclonal | 1:50 in TBS + 0,1% Tween, permeabilization in 0,1% Triton X in TBS |
| PIWIL1 | | Invitrogen, MA5-41250, rabbit, polyclonal; epitope aa733-831 | 1:200 in blocking solution (25% goat serum in 0,5% BSA/TBS) |
| GTSF1 | | ATLAS antibodies, HPA038876, rabbit, polyclonal | 1:50 in 0,5% BSA/TBS |
| TDRD1 | | Antibodies.com, A54784, rabbit, polyclonal | 1:100 in 5% BSA/TBS |
| HENMT1 | | Invitrogen, PA5-55866, rabbit, polyclonal | 1:150 in blocking solution (25% goat serum in 5% BSA/TBS) |
| GPAT2 | | Sigma Aldrich, HPA036841, rabbit, polyclonal | 1:50 in blocking solution (25% goat serum in 5% BSA/TBS) |
| DDX4 | | Abcam, ab13840, rabbit, polyclonal | 1:2000 in 5% BSA/TBS |
| MAEL | | Novus Biologicals, NBP2-69070, rabbit, polyclonal | 1:300 in 5% BSA/TBS |
| LINE-1 ORF1p | | Abcam, ab245249, rabbit, monoclonal | 1:100 in blocking solution (25% goat serum in 5% BSA/TBS) |
| | | Abcam, ab230966, rabbit, monoclonal | 1:200 -1:500 in blocking solution (25% goat serum in 5% BSA/TBS) |
| PLD6 | | Novus Biologicals, NBP2-13771, rabbit, polyclonal | 1:100 in blocking solution (25% goat serum in 5% BSA/TBS) |
| GAPDH | WB | Cell signalling, 5174, rabbit, monoclonal | 1:1500 in blocking solution (5% milk powder in TBST) |
| HA-tag | | Roche Sigma, 11867423, rat monoclonal, | 1:2000 in blocking solution (5% milk powder in TBST) |

## Supplementary Figures

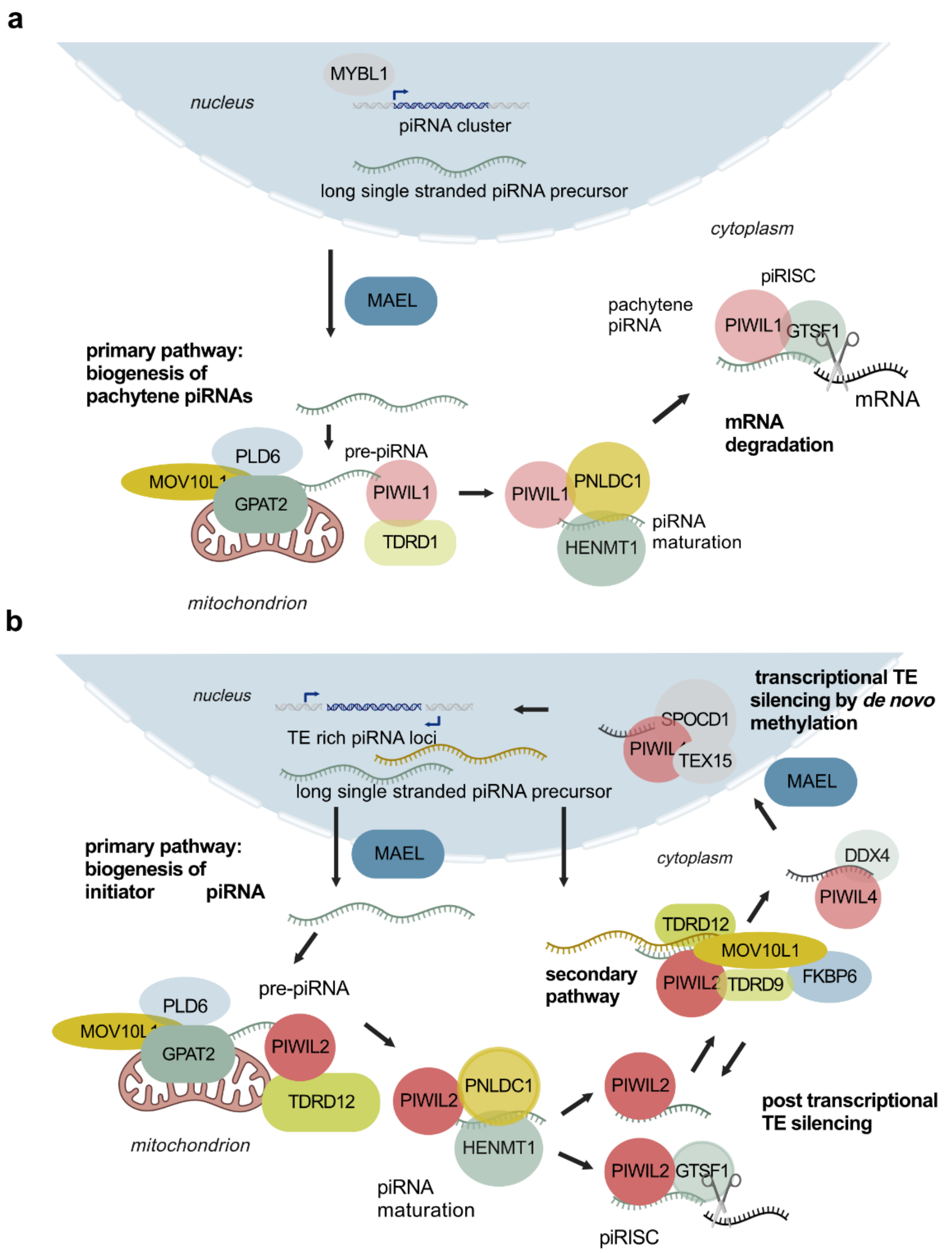


**Supplementary Figure 1. Schematic overview on piRNA biogenesis in the mammalian testis.** a. In the adult mammalian testis, the generation of pachytene piRNAs is restricted to the primary pathway where long non-TE derived piRNA precursors are transported from the nucleus to the cytoplasm and accumulate in perinuclear structures called nuage. Here, they are cleaved by the endonuclease PLD6, which establishes the 5'-ends of pre-piRNAs[5,30]. Activity of PLD6 depends on RNA helicase MOV10L1 that binds to piRNA precursors and feeds them to PLD6. In addition, GPAT2, which is also located at the outer membrane of mitochondria, is suspected to be involved in this process. The cleaved pre-piRNAs are then loaded onto PIWIL1 or PIWIL2 for further 3'-end maturations in which PNLDC1 trims the piRNA precursor RNA to its final length and HENMT1 adds the 2′-O-methyl group to the 5'-end[31]. PIWIL1 selectively binds to pachytene piRNAs and the activity of the formed piRNA induced silencing (piRISC) complex depends on the binding of the enhancer protein GTSF1[32]. b. In fetal germ cells, the secondary biogenesis pathway enables massive amplification of TE derived piRNAs. Here, PIWIL2 containing piRISC complexes slice the complementary long piRNA precursor transcripts to generate pre-piRNAs with a monophosporylated 5'-end[33]. In addition to the factors described above, the functions of further proteins, such as the scaffold Tudor domain-containing (TDRD) proteins, RNA helicase DDX4 and nucleo-cytoplasmic shuttling protein MAEL are indispensable for the piRNA pathway. Supplementary Figure 1a,b created with BioRender.com released under a Creative Commons Attribution-NonCommercial-NoDerivs 4.0 International license (https://creativecommons.org/licenses/by-nc-nd/4.0/deed.en).

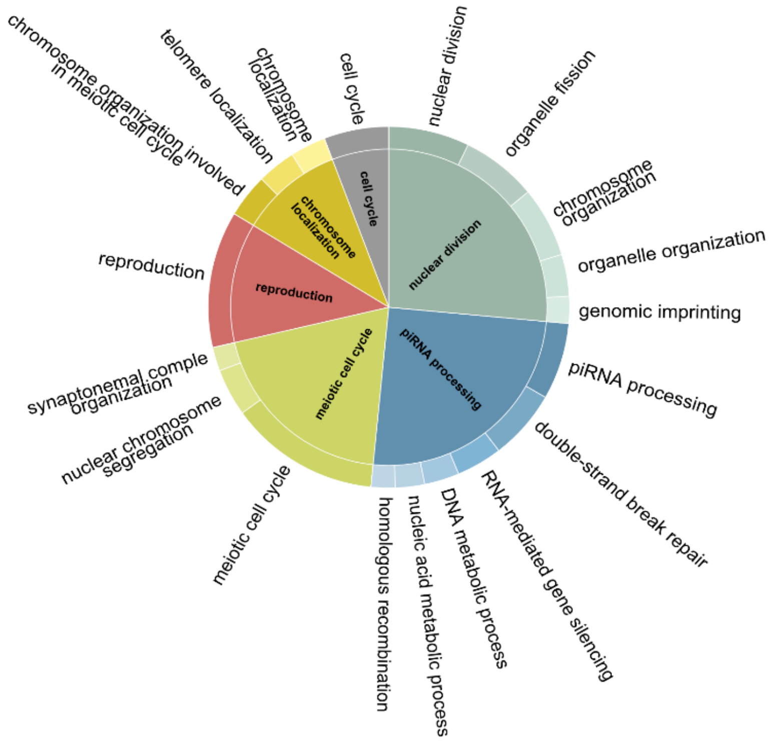


**Supplementary Figure 2.** Pie chart illustrating two-tiered biological sub-processes clustering to main biological processes identified in Gene Ontology (GO) analysis.

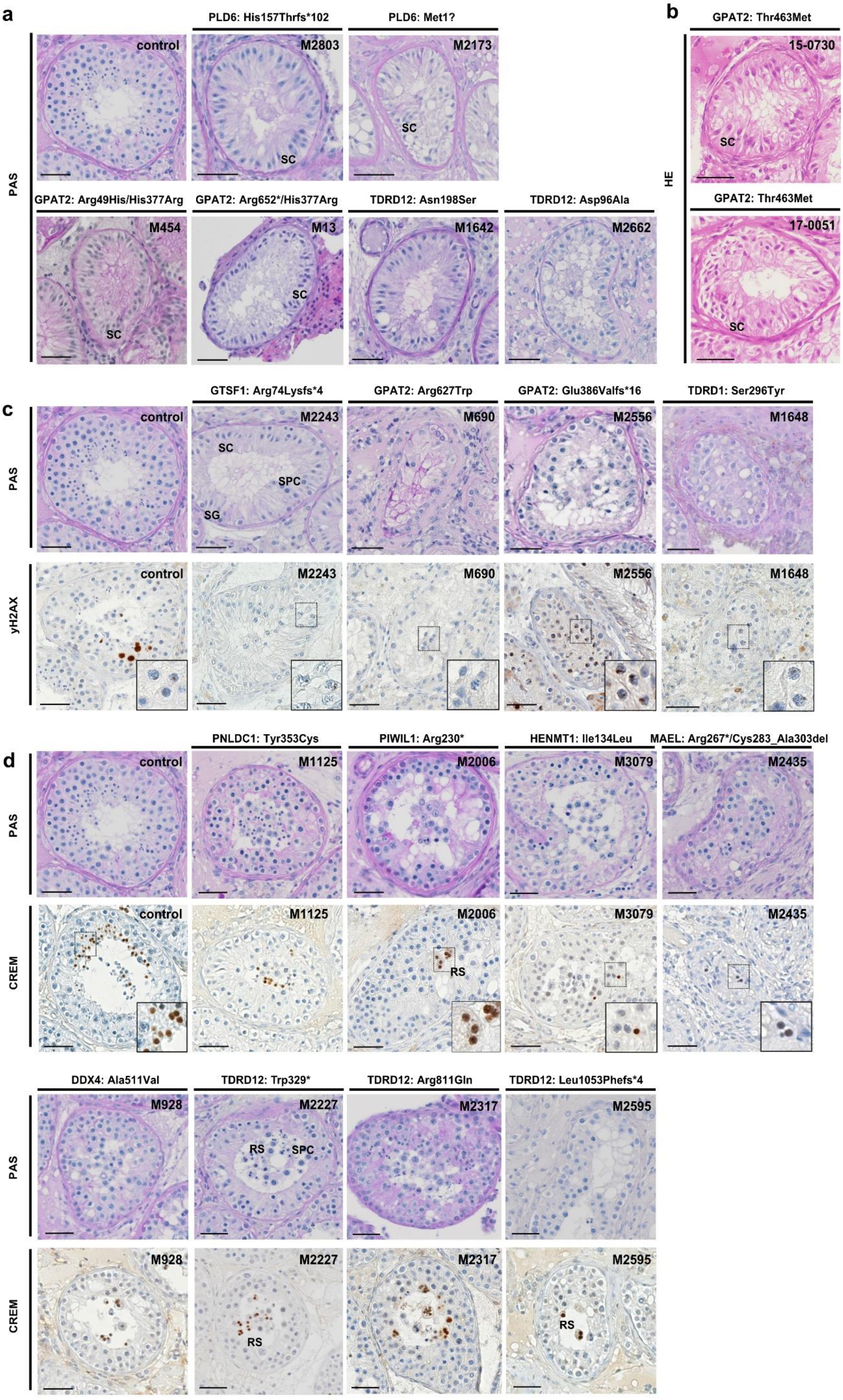
a
PLD6: His157Thrfs*102
PLD6: Met1?
control
M2803
M2173
SC
SC
PAS
GPAT2: Arg49His/His377Arg
GPAT2: Arg652*/His377Arg
TDRD12: Asn198Ser
TDRD12: Asp96Ala
M454
M13
M1642
M2662
SC
SC
b
GPAT2: Thr463Met
15-0730
SC
HE
GPAT2: Thr463Met
17-0051
SC
c
GTSF1: Arg74Lysfs*4
GPAT2: Arg627Trp
GPAT2: Glu386Valfs*16
TDRD1: Ser296Tyr
control
M2243
M690
M2556
M1648
SC
SPC
SG
PAS
γH2AX
d
PNLDC1: Tyr353Cys
PIWIL1: Arg230*
HENMT1: Ile134Leu
MAEL: Arg267*/Cys283_Ala303del
control
M1125
M2006
M3079
M2435
PAS
CREM
RS
DDX4: Ala511Val
TDRD12: Trp329*
TDRD12: Arg811Gln
TDRD12: Leu1053Phefs*4
M928
M2227
M2317
M2595
RS
SPC
PAS
CREM
RS
RS

**Supplementary Figure 3. Testicular phenotypes of patients with biallelic high impact variants in genes of the piRNA pathway.** a. Periodic acid–Schiff (PAS) staining demonstrating absence of germ cells (SCO) in M2803, M2173, M454, M13, M1642 and M2662. b. Hematoxylin-Eosin (HE) staining demonstrating SCO in 15-0730 and 17-051. c. PAS staining demonstrating meiotic arrest (MeiA) in M2243, M690, M2556, and M1648. Representative tubules show the staining pattern observed in independent sections (control: N = 3, proband: N = 2) are shown. γH2AX positive sex bodies indicative for pachytene spermatocytes are present in seminiferous tubules of most patients with MeiA (control: N = 3, proband: N = 1). d. PAS staining demonstrating presence of haploid germ cells in seminiferous tubules of M1125, M2006, M3079, M2435, M928, M2227, M2317, and M2595. Representative tubules show the most advanced stage of spermatogenesis observed in 3 independent sections are shown. Cyclic AMP Element Modulator (CREM) positive round spermatids are present in seminiferous tubules (control: N = 3, proband: N = 1). Scale bar = 50 µm. SC: Sertoli cell; SG: spermatogonia; SPC: spermatocyte; RS: round spermatid.

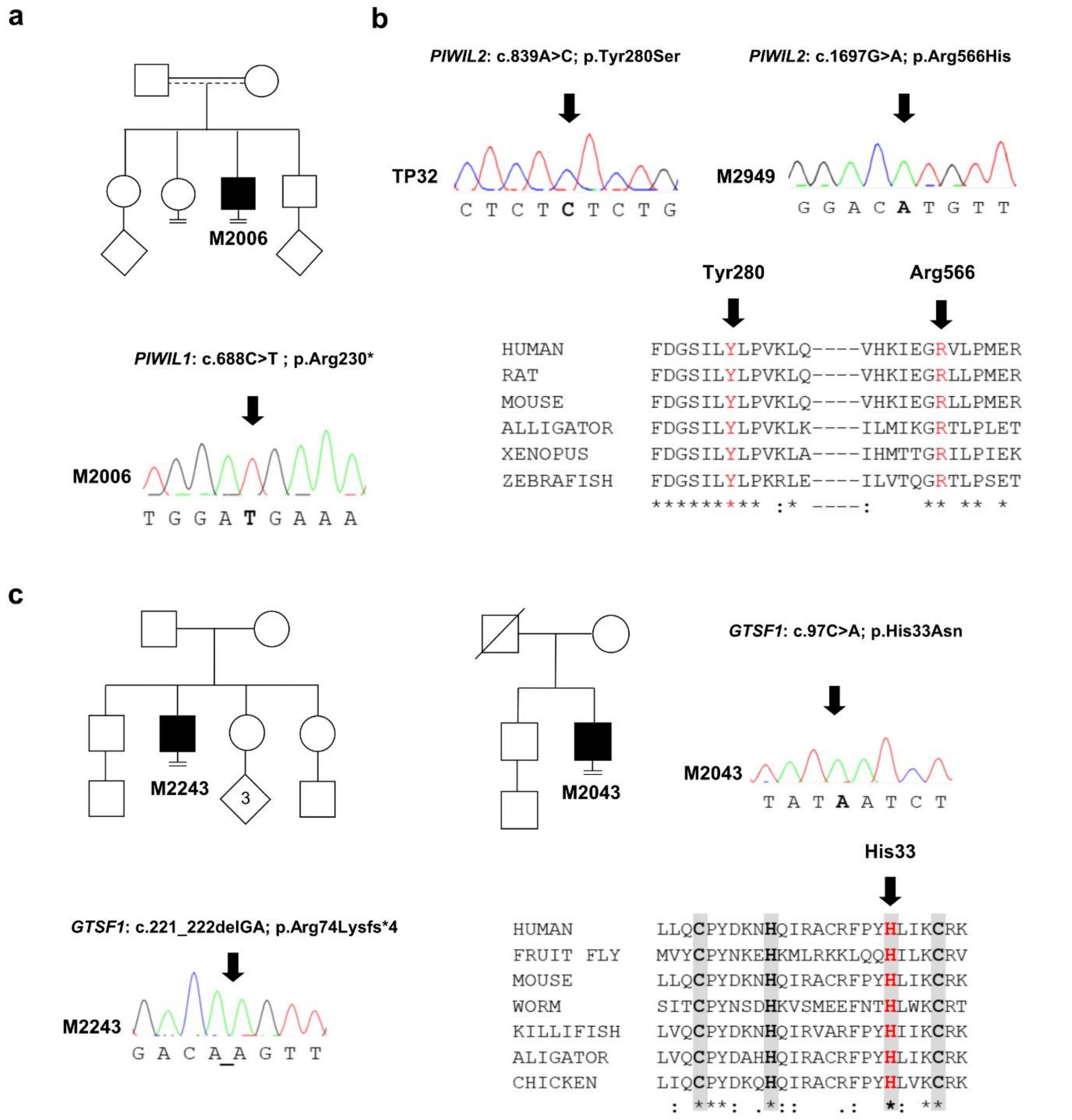


**Supplementary Figure 4. Genetic data on homozygous high impact variants in *PIWIL1*, *PIWIL2* and *GTSF1*.** a. Family pedigree of M2006 and Sanger traces of homozygous *PIWIL1* variant c.688C>T p.(Arg230*). b. Sanger traces of *PIWIL2* variants c.839A>C p.(Tyr280Ser) and c.1697G>A p.(Arg566His). Multiple sequence alignment of human PIWIL2 and orthologues demonstrating conservation of both mutated amino acid residues up to zebrafish. c. Family pedigree of M2243 and M2043 with homozygous frameshift variant c.221_222delGA p.(Arg74Lysfs*4) and missense variant c.97C>A p.(His33Asn) in *GTSF1*, respectively. Multiple sequence alignment of human GTSF1 and orthologues demonstrating conservation of the first GTSF1 zinc finger domain (grey bars). Histidin 33 is indicated in red. * identical amino acid residue in all orthologous proteins, : exchange to highly similar amino acid residue tolerated.

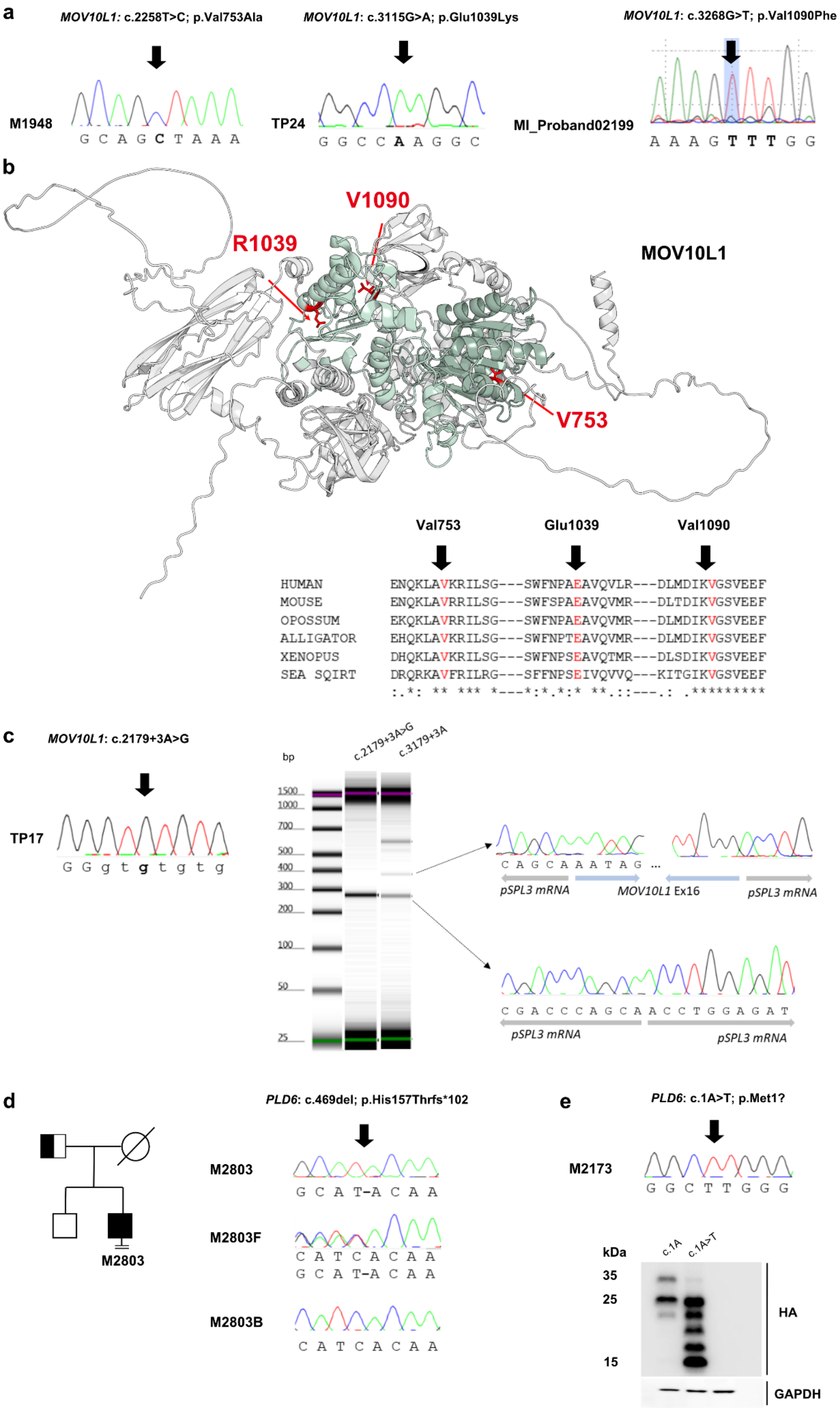
a
MOV10L1: c.2258T>C; p.Val753Ala
MOV10L1: c.3115G>A; p.Glu1039Lys
MOV10L1: c.3268G>T; p.Val1090Phe
M1948
G C A G C T A A A
TP24
G G C C A A G G C
MI_Proband02199
A A A G T T T G G
b
V1090
R1039
MOV10L1
V753
Val753
Glu1039
Val1090
HUMAN ENQKLAVKRILSG---SWFNPAEAVQVLR---DLMDIKVGSVEEF
MOUSE ENQKLAVRRILSG---SWFSPAEAVQVMR---DLTDIKVGSVEEF
OPOSSUM EKQKLAVRRILSG---SWFNPAEAVQVMR---DLMDIKVGSVEEF
ALLIGATOR EHQKLAVKRILSG---SWFNPTEAVQVMR---DLMDIKVGSVEEF
XENOPUS DHQKLAVKRILSG---SWFNPSEAVQTMR---DLSDIKVGSVEEF
SEA SQIRT DRQRKAVFRILRG---SFFNPSEIVQVVQ---KITGIKVGSVEEF
c
MOV10L1: c.2179+3A>G
TP17
G G g t g t g t g
bp
c.2179+3A>G
c.3179+3A
1500
1000
700
500
400
300
200
100
50
25
C A G C A A A T A G ...
pSPL3 mRNA
MOV10L1 Ex16
pSPL3 mRNA
C G A C C C A G C A A C C T G G A G A T
pSPL3 mRNA
pSPL3 mRNA
d
PLD6: c.469del; p.His157Thrfs*102
M2803
G C A T-A C A A
M2803F
C A T C A C A A
G C A T-A C A A
M2803B
C A T C A C A A
M2803
e
PLD6: c.1A>T; p.Met1?
M2173
G G C T T G G G
kDa
c.1A
c.1A>T
35
25
15
HA
GAPDH

**Supplementary Figure 5. Genetic data on homozygous high impact variants in *MOV10L1* and *PLD6*.** a. Sanger traces of homozygous *MOV10L1* variants c.2258T>C p.(Val753Ala) in M1948, c.3115G>A p.(Glu1039Lys) in TP24 and c.3268G>T p.(Val1090Phe) in MI_Proband021994. b. AlphaFold2-predicted structure of MOV10L1 with helicase domains coloured (green:DNA2/NAM7 helicase; light green: DNA2/NAM7 helicase-like) and amino acid residues affected by homozygous substitutions highlighted in red; multiple sequence alignment of human MOV10L1 and orthologous proteins. Affected valine residues 753 and 1090 as well as glutamine 1039 in human MOV10L1 are conserved up to sea squirt. c. Sanger traces showing homozygous *MOV10L1* variant c.2179+3A>G in TP17. Minigene assay demonstrating skipping of *MOV10L1* exon 16 in c.2179+3A>G. As a result, 109 base pairs are deleted from the open reading frame and a premature stop codon is introduced at position 691, p.(Asn691*). Source data are provided as a Source Data file. d. Pedigree information on M2803 with homozygous frameshift variant c.469del in *PLD6*. Sanger traces of *PLD6* reveal presence of c.469del in the homozygous state in the index case (M2803) and in the heterozygous state in the father (M3803F), whereas the unaffected brother carries the wild type nucleotide on both alleles. e. Sanger traces showing homozygous *PLD6* start loss variant c.1A>T (p.Met1?) in M2173. Western blot analysis of HA-tagged PLD6 derived from wildtype and mutant (*PLD6*:c.1A>T) transcripts. In the mutant translation at the original start codon is abolished, demonstrated by a strikingly reduced amount of PLD6 protein with a calculated molecular weight of 35 kDa. In contrast translation initiation starts form an in-frame translation initiation site at position c.385 and further in-frame ATGs downstream, resulting in truncated PLD6 proteins lacking 129 or more amino acids at the N-terminus. Representative blot of three replicates is shown. Source data are provided as a Source Data file.

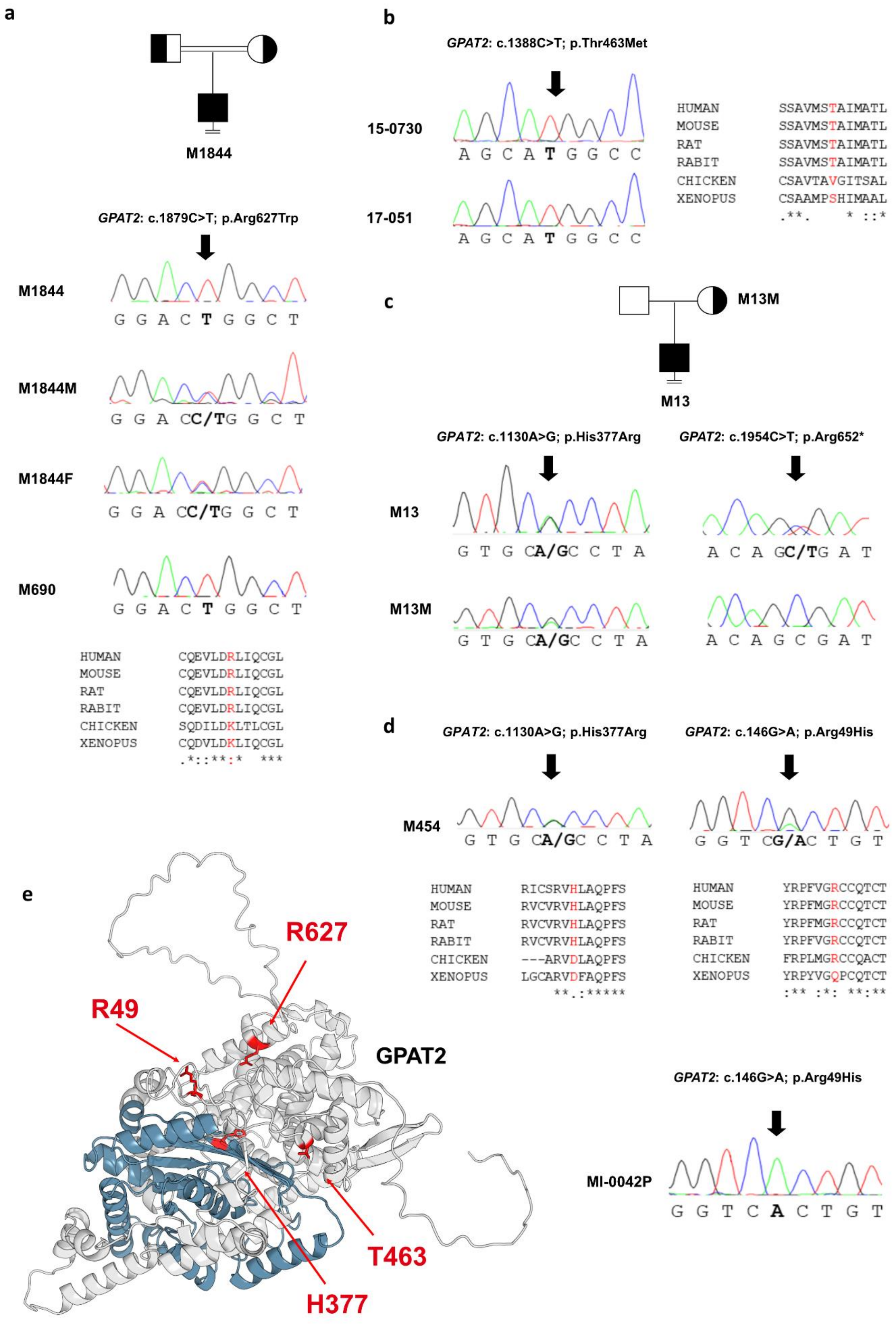
a
M1844
GPAT2: c.1879C>T; p.Arg627Trp
M1844
G G A C T G G C T
M1844M
G G A CC/TG G C T
M1844F
G G A CC/TG G C T
M690
G G A C T G G C T
HUMAN CQEVLDRLIQCGL
MOUSE CQEVLDRLIQCGL
RAT CQEVLDRLIQCGL
RABIT CQEVLDRLIQCGL
CHICKEN SQDILDKLTLCGL
XENOPUS CQDVLDKLIQCGL
b
GPAT2: c.1388C>T; p.Thr463Met
15-0730
A G C A T G G C C
17-051
A G C A T G G C C
HUMAN SSAVMSTAIMATL
MOUSE SSAVMSTAIMATL
RAT SSAVMSTAIMATL
RABIT SSAVMSTAIMATL
CHICKEN CSAVTAVGITSAL
XENOPUS CSAAMPSHIMAAL
c
M13M
M13
GPAT2: c.1130A>G; p.His377Arg
GPAT2: c.1954C>T; p.Arg652*
M13
G T G CA/GC C T A
A C A GC/TG A T
M13M
G T G CA/GC C T A
A C A G C G A T
d
GPAT2: c.1130A>G; p.His377Arg
GPAT2: c.146G>A; p.Arg49His
M454
G T G CA/GC C T A
G G T CG/AC T G T
HUMAN RICSRVHLAQPFS
MOUSE RVCVRVHLAQPFS
RAT RVCVRVHLAQPFS
RABIT RVCVRVHLAQPFS
CHICKEN ---ARVDLAQPFS
XENOPUS LGCARVDFAQPFS
HUMAN YRPFVGRCCQTCT
MOUSE YRPFMGRCCQTCT
RAT YRPFMGRCCQTCT
RABIT YRPFVGRCCQTCT
CHICKEN FRPLMGRCCQACT
XENOPUS YRPYVGQPCQTCT
GPAT2: c.146G>A; p.Arg49His
MI-0042P
G G T C A C T G T
e
R627
R49
GPAT2
T463
H377

**Supplementary Figure 6. Genetic data on biallelic high impact variants identified in *GPAT2.*** a. Sanger traces of *GPAT2* variant c.1879C>T p.(Arg627Trp) identified in the homozygous state in M1844 and M690. Parents of M1844 carry the variant in the heterozygous state. Multiple sequence alignment of GPAT2 orthologous proteins revealing conservation of Arg627 up to *Xenopus laevis*. b. Sanger traces of the homozygous *GPAT2* missense variant c.1388C>T p.(Thr463Met) identified in 15-0730 and 17-051. Threonin 463 in human GPAT2 is conserved among mammals. c. Sanger traces of heterozygous *GPAT2* variants c.1130A>G p.(His377Arg) and c.1954C>T p.(Arg652*) identified in M13. The mother of M13 only carried variant c.1130A>G. d. M454 reveals compound heterozygous setting (confirmed by long read sequencing) of heterozygous *GPAT2* variants c.1130A>G p.(His377Arg) and c.146G>A p.(Arg49His). Multiple sequence alignment of GPAT2 orthologous proteins demonstrating conservation of Arg46 and His377. Sanger traces showing homozygous *GPAT2* variant c.146G>A p.(Arg49His) in MI-0042P. e. AlphaFold2-predicted structure of GPAT2 with GPAT/DHAPAT acetyltransferase domain depicted in blue and amino acid residues affected by novel missense variants highlighted in red. * identical amino acid residue in all orthologous proteins, : exchange to highly similar amino acid residue tolerated.

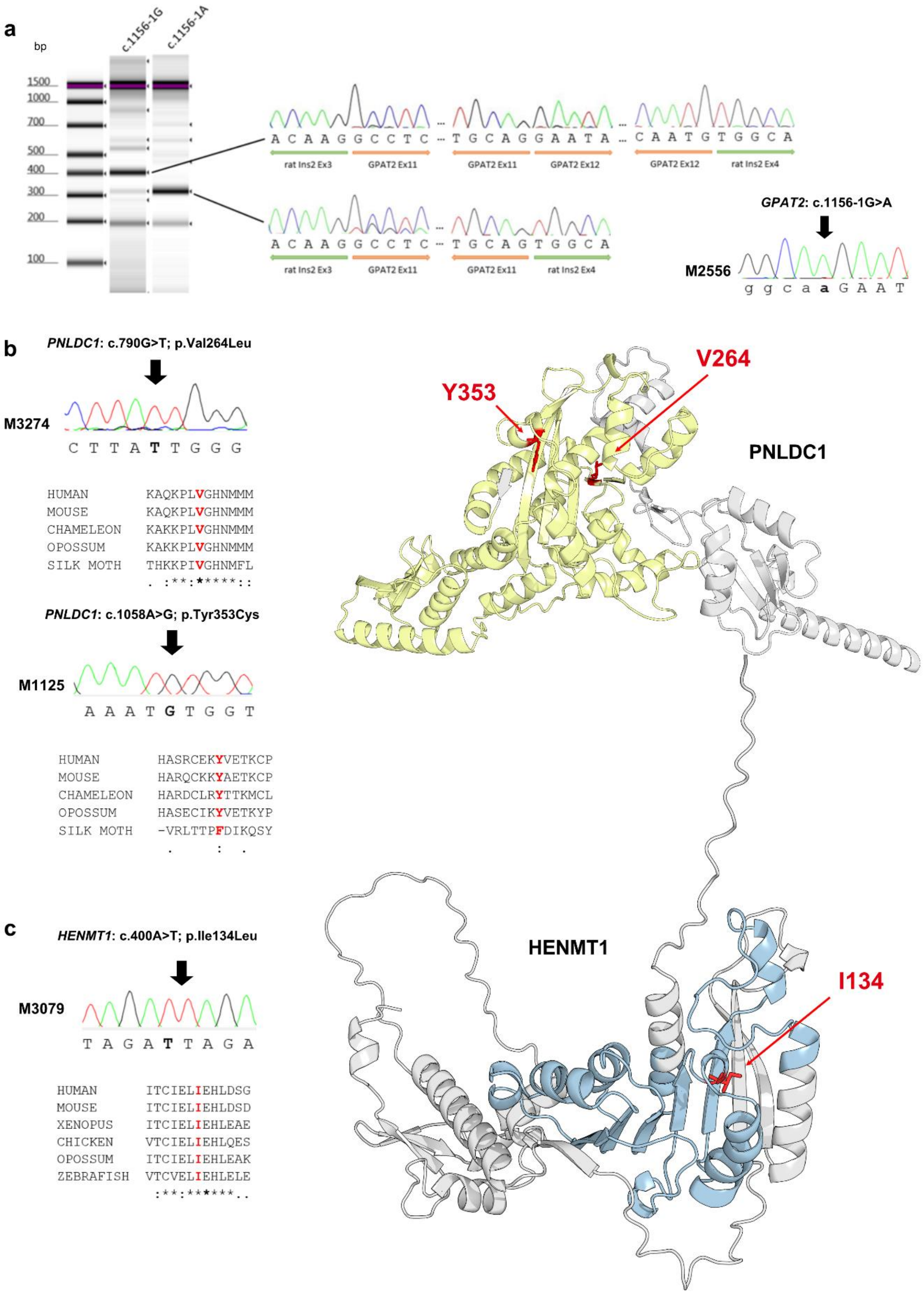
a
bp
c.1156-1G
c.1156-1A
1500
1000
700
500
400
300
200
100
A C A A G G C C T C ··· T G C A G G A A T A ... C A A T G T G G C A
rat Ins2 Ex3
GPAT2 Ex11
GPAT2 Ex11
GPAT2 Ex12
GPAT2 Ex12
rat Ins2 Ex4
A C A A G G C C T C ··· T G C A G T G G C A
rat Ins2 Ex3
GPAT2 Ex11
GPAT2 Ex11
rat Ins2 Ex4
GPAT2: c.1156-1G>A
M2556
g g c a a G A A T
b
PNLDC1: c.790G>T; p.Val264Leu
M3274
C T T A T T G G G
HUMAN KAQKPLVGHNMMM
MOUSE KAQKPLVGHNMMM
CHAMELEON KAKKPLVGHNMMM
OPOSSUM KAKKPLVGHNMMM
SILK MOTH THKKPIVGHNMFL
. :**:*****::
PNLDC1: c.1058A>G; p.Tyr353Cys
M1125
A A A T G T G G T
HUMAN HASRCEKYVETKCP
MOUSE HARQCKKYAETKCP
CHAMELEON HARDCLRYTTKMCL
OPOSSUM HASECIKYVETKYP
SILK MOTH -VRLTTPFDIKQSY
. : .
c
HENMT1: c.400A>T; p.Ile134Leu
M3079
T A G A T T A G A
HUMAN ITCIELIEHLDSG
MOUSE ITCIELIEHLDSD
XENOPUS ITCIELIEHLEAE
CHICKEN VTCIELIEHLQES
OPOSSUM ITCIELIEHLEAK
ZEBRAFISH VTCVELIEHLELE
:**:*****..
Y353
V264
PNLDC1
HENMT1
I134

**Supplementary Figure 7. Genetic data on biallelic high impact variants identified in *GPAT2*, *PNLDC1* and *HENMT1*.** a. Sanger traces of homozygous *GPAT2* splice site variant c.1156-1G>A identified in M2556. The variant leads to skipping of *GPAT2* exon 12 in minigene splice assay. This results in a frameshift of the open reading frame and subsequent insertion of a premature stop codon, p.(Glu386Valfs*16), in case the mutant transcript is not degraded by NMD. Source data are provided as a Source Data file. b. Sanger traces demonstrating homozygous variants c.790G>T and c.1058A>G in *PNLDC1* in M3274 and M1125, respectively; multiple sequence alignment of PNLDC1 orthologous proteins. Affected valine 264 and tyrosine 353 are inducted in red. AlphaFold2-predicted structure with amino acid residues affected by homozygous substitutions depicted in red and Ribonuclease CAF1 domain highlighted in yellow. c. Sanger traces demonstrating homozygous missense variant c.400A>T in *HENMT1* in M3079; multiple sequence alignment of HENMT1 orthologous proteins and AlphaFold2-predicted structure with affected residue isoleucine 134 depicted in red and Methyltransferase domain indicated in blue.* identical amino acid residue in all orthologous proteins, : exchange to highly similar amino acid residue tolerated.

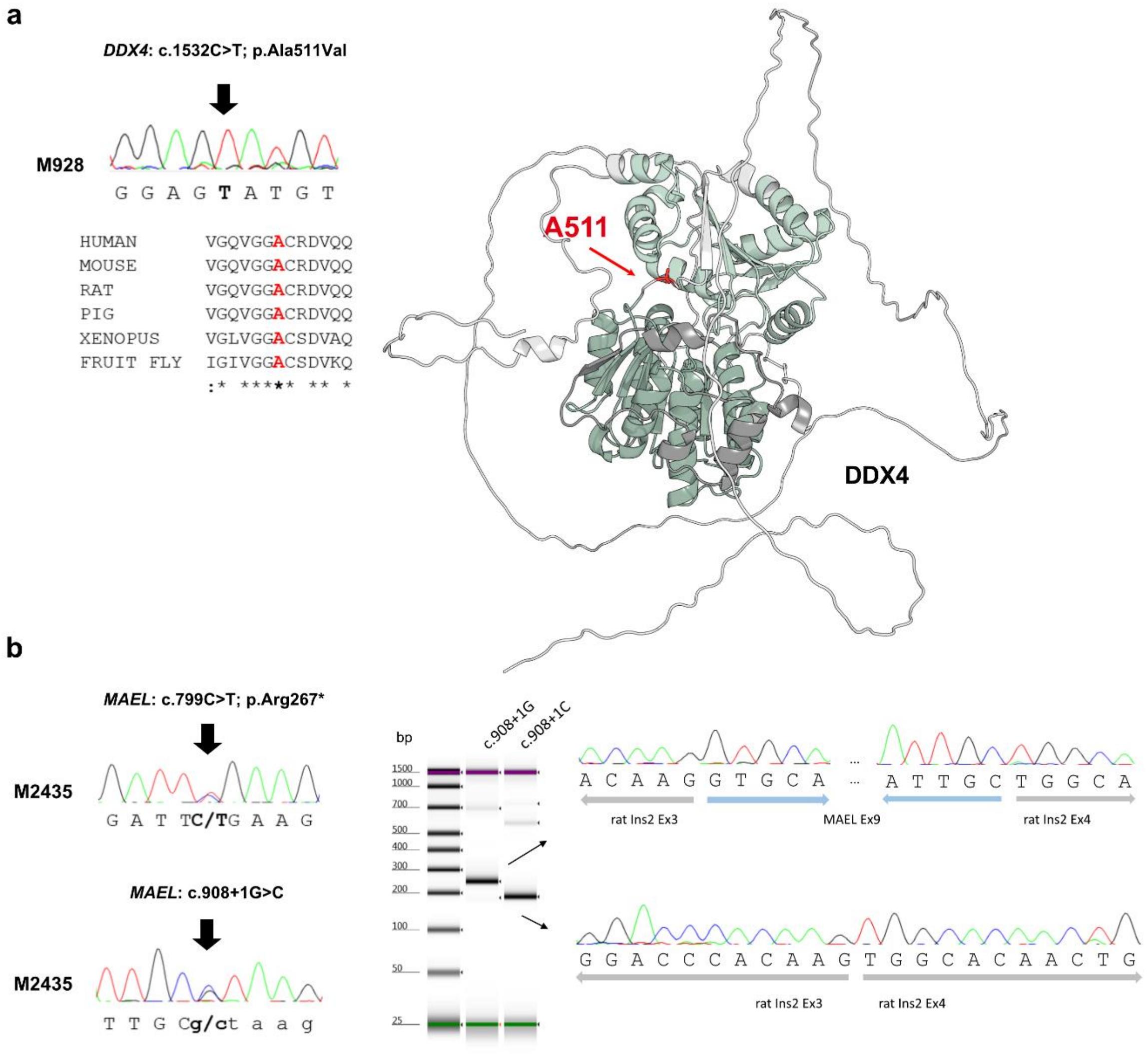


**Supplementary Figure 8. Genetic data on biallelic high impact variants identified in *DDX4* and *MAEL*.** a. Sanger traces demonstrating homozygous variant c.1532C>T in *DDX4* in M928; multiple sequence alignment of DDX4 orthologous proteins and AlphaFold2-predicted protein structure with affected residue alanine 51 depicted in red. In the protein structure domains are coloured (grey: Q motif, dark green: Helicase domains). b. Sanger traces showing heterozygous variants c.799C>T and c.908+1G>C in *MAEL* in M2435. Compound heterozygosity of both variants was confirmed by long read sequencing. In minigene assay c.908+1G>C results in skipping of *MAEL* exon 9, leading to an in-frame deletion of 21 amino acids p.(Cys283_Ala303del). Source data are provided as a Source Data file.

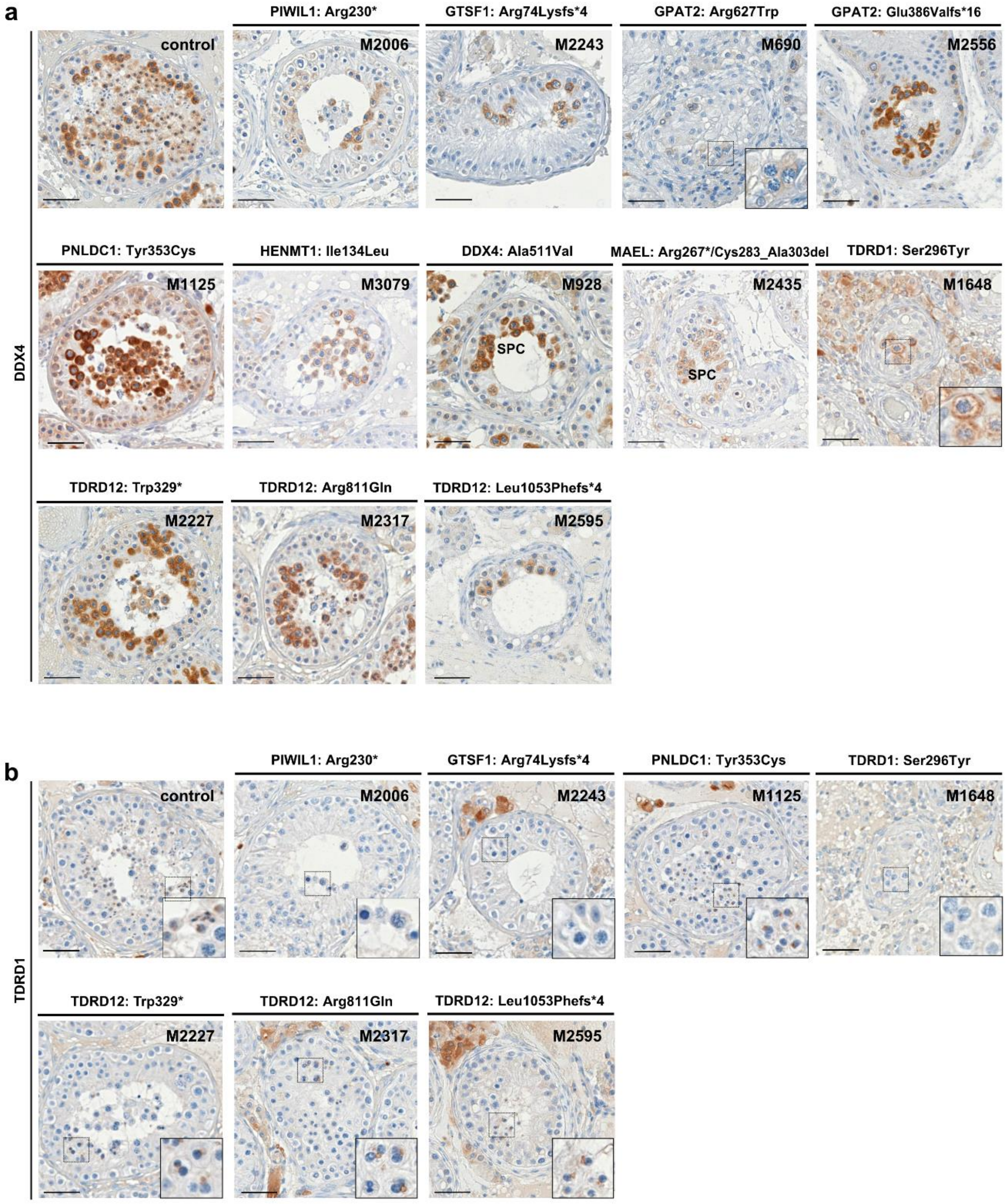


**Supplementary Figure 9. Testicular expression profile of piRNA pathway components DDX4 and TDRD1 in control and identified variant carriers.** a. Immunohistochemical (IHC) staining for DDX4 in testicular tissue of men with full spermatogenesis (control) and variant carriers. b. IHC staining for TDRD1 in testicular tissue of men with full spermatogenesis (control) and variant carriers**.** Representative tubules show the staining pattern observed in independent sections (control: N = 3, proband: N = 2 in case the staining pattern differed from the control) are shown. Scale bar = 50 µm.SPC: spermatocyte.

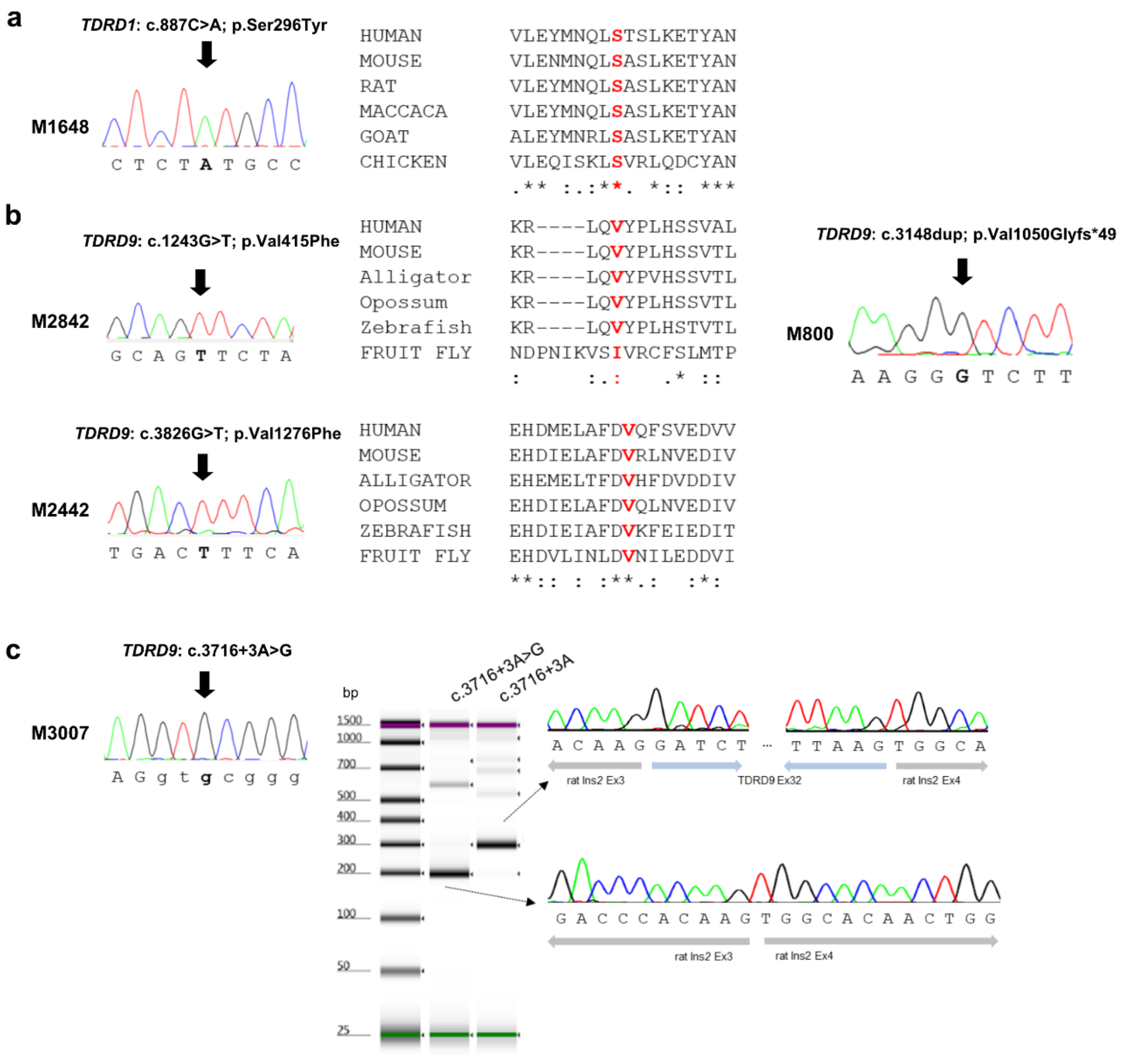


**Supplementary Figure 10. Genetic data on biallelic high impact variants identified in *TDRD1* and *TDRD9*.** a. Sanger traces demonstrating homozygous variant c.887C>A in *TDRD1* in M1648; multiple sequence alignment of TDRD1 orthologues. Affected serine residue is highlighted in red. b. Sanger traces demonstrating homozygous variants c.1243G>T, c.3148dup and c.3826G>T in *TDRD9* in M2842, M800 and M2442, respectively; multiple sequence alignment of TDRD9 orthologues, affected valine residues in red. c. Sanger traces of homozygous *TDRD9* splice region variant c.3716+3A>G in M3007; minigene assay results of c.3716+3A>G showing skipping of *TDRD9* exon 32. This results in a frameshift and insertion of a stop codon p.(Ser1208Leufs*56), if the transcript is not degraded by NMD. Source data are provided as a Source Data file. *identical amino acid residue in all orthologous proteins, : exchange to highly similar amino acid residue tolerated.

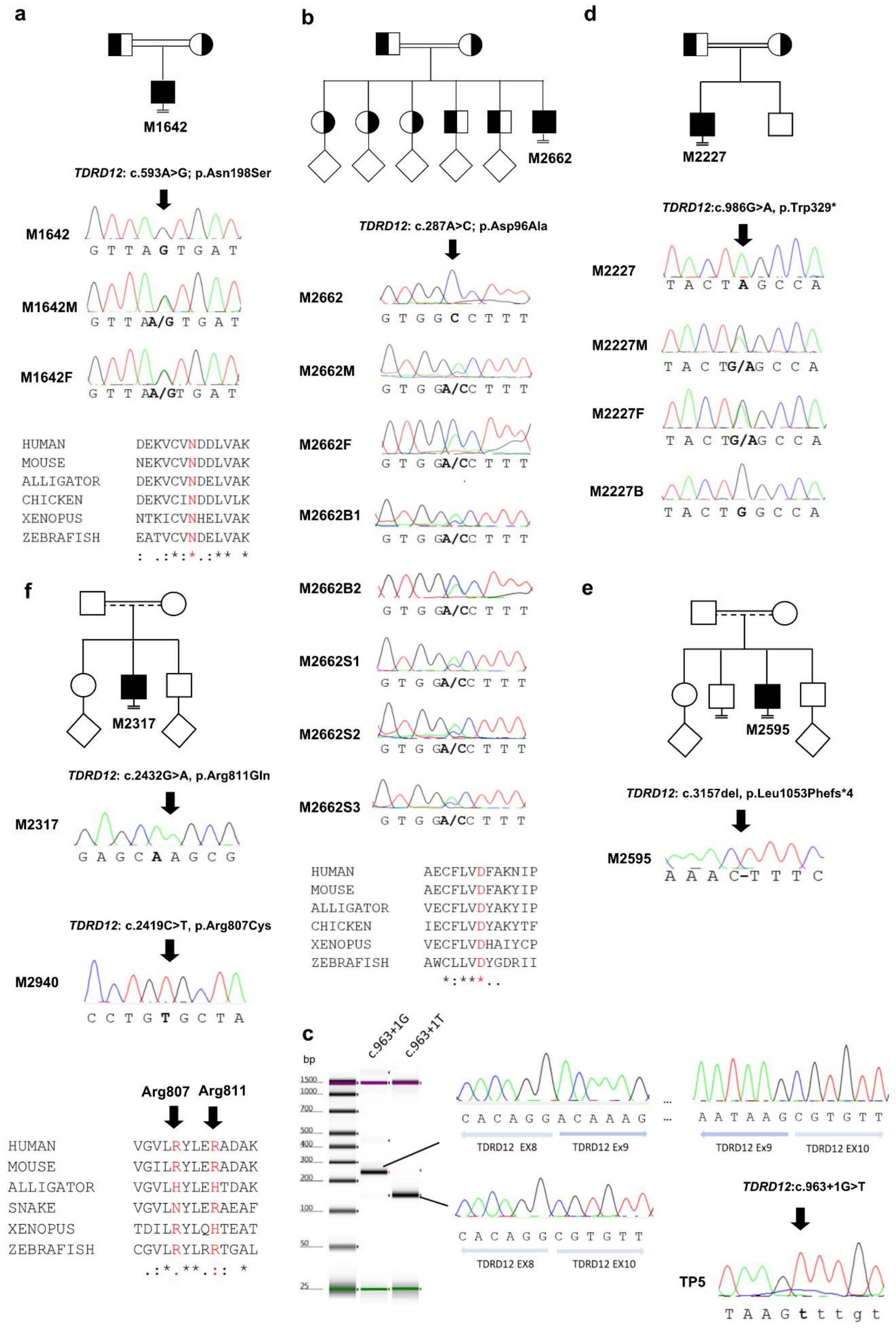
a
M1642
TDRD12: c.593A>G; p.Asn198Ser
M1642
G T T A G T G A T
M1642M
G T T AA/G T G A T
M1642F
G T T AA/GT G A T
HUMAN DEKVCVNDDLVAK
MOUSE NEKVCVNDDLVAK
ALLIGATOR DEKVCVNDELVAK
CHICKEN DEKVCINDDLVLK
XENOPUS NTKICVNHELVAK
ZEBRAFISH EATVCVNDELVAK
b
M2662
TDRD12: c.287A>C; p.Asp96Ala
M2662
G T G G C C T T T
M2662M
M2662F
M2662B1
M2662B2
M2662S1
M2662S2
M2662S3
G T G GA/CC T T T
HUMAN AECFLVDFAKNIP
MOUSE AECFLVDFAKYIP
ALLIGATOR VECFLVDYAKYIP
CHICKEN IECFLVDYAKYTF
XENOPUS VECFLVDHAIYCP
ZEBRAFISH AWCLLVDYGDRII
d
M2227
TDRD12:c.986G>A, p.Trp329*
M2227
T A C T A G C C A
M2227M
T A C TG/AG C C A
M2227F
T A C TG/AG C C A
M2227B
T A C T G G C C A
e
M2595
TDRD12: c.3157del, p.Leu1053Phefs*4
M2595
A A A C-T T T C
f
M2317
TDRD12: c.2432G>A, p.Arg811Gln
M2317
G A G C A A G C G
TDRD12: c.2419C>T, p.Arg807Cys
M2940
C C T G T G C T A
Arg807 Arg811
HUMAN VGVLRYLERADAK
MOUSE VGILRYLERADAK
ALLIGATOR VGVLHYLEHTDAK
SNAKE VGVLNYLERAEAF
XENOPUS TDILRYLQHTEAT
ZEBRAFISH CGVLRYLRRTGAL
c
c.963+1G
c.963+1T
bp
1500
1000
700
500
400
300
200
100
50
25
C A C A G G A C A A A G
TDRD12 EX8
TDRD12 Ex9
A A T A A G C G T G T T
TDRD12 Ex9
TDRD12 EX10
C A C A G G C G T G T T
TDRD12 EX8
TDRD12 EX10
TDRD12:c.963+1G>T
TP5
T A A G t t t g t

**Supplementary Figure 11. Genetic data on biallelic high impact variants identified in *TDRD12*.** a. Pedigree information on M1642 with homozygous missense variant c.593A>G in *TDRD12*. Sanger traces reveal presence of c.593A>G in homozygous state in the index case and in heterozygous state in both parents; multiple sequence alignment of TDRD12 orthologues, affected asparagine residue 198 in red. *TDRD12* variant c.593A>G has already been described[34]. b. Pedigree of M2662; variant c.287A>C is present in homozygous state in the infertile index case. All fertile siblings of M2662 as well as both parents are heterozygous carriers of c.287A>C; multiple sequence alignment of TDRD12 orthologues with affected aspartate 96 highlighted in red. c. Minigene assay demonstrating skipping of *TDRD12* exon 9 in c.963+1G>T, resulting in a frameshift, p.(Asp289Alafs*3), if the mutant transcript is not degraded by NMD. Source data are provided as a Source Data file. Sanger traces showing homozygous variant c.963+1G>T in *TDRD12* in TP5. d. Pedigree of M2227 with homozygous stop-gain variant p.(Trp329*) in *TDRD12.* The variant is inherited by both parents and absent in the brother. A sister who obtained the diagnosis of premature ovarian insufficiency is also carrying p.(Trp329*) in the homozygous state. e. Pedigree of M2595 with homozygous frameshift variant c.3157del p.(Leu1053Phefs*4) in *TDRD12*. The patient has an infertile brother of whom DNA material was not available. f. Sanger traces demonstrating homozygous missense variants c.2432G>A p.(Arg811Gln) and c.2419C>T p.(Arg807Cys) in *TDRD12*. Multiple sequence alignment of TDRD12 orthologues, affected arginine residues 807 and 811 are highlighted in red. *identical amino acid residue in all orthologous proteins, : exchange to highly similar amino acid residue tolerated.

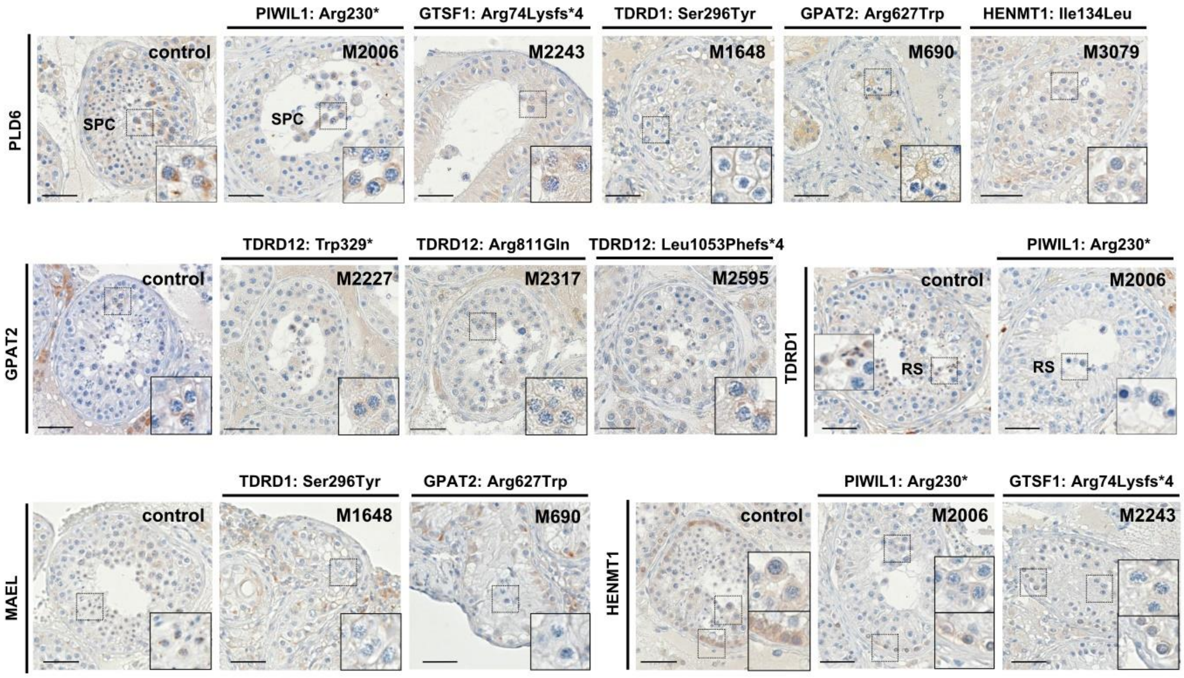


**Supplementary Figure 12. Expression of key piRNA pathway components in testicular tissue of variant carriers.** Immunohistochemical staining for piRNA pathway associated proteins demonstrating diminished expression of PLD6, GPAT2, TDRD1, MAEL and HENMT1 in patients with biallelic high impact variants in piRNA pathway genes. Representative tubules show the staining pattern observed in independent sections (control: N = 3, proband: N = 2) are shown. Scale bar = 50 µm. SPC: spermatocyte; RS: round spermatid.

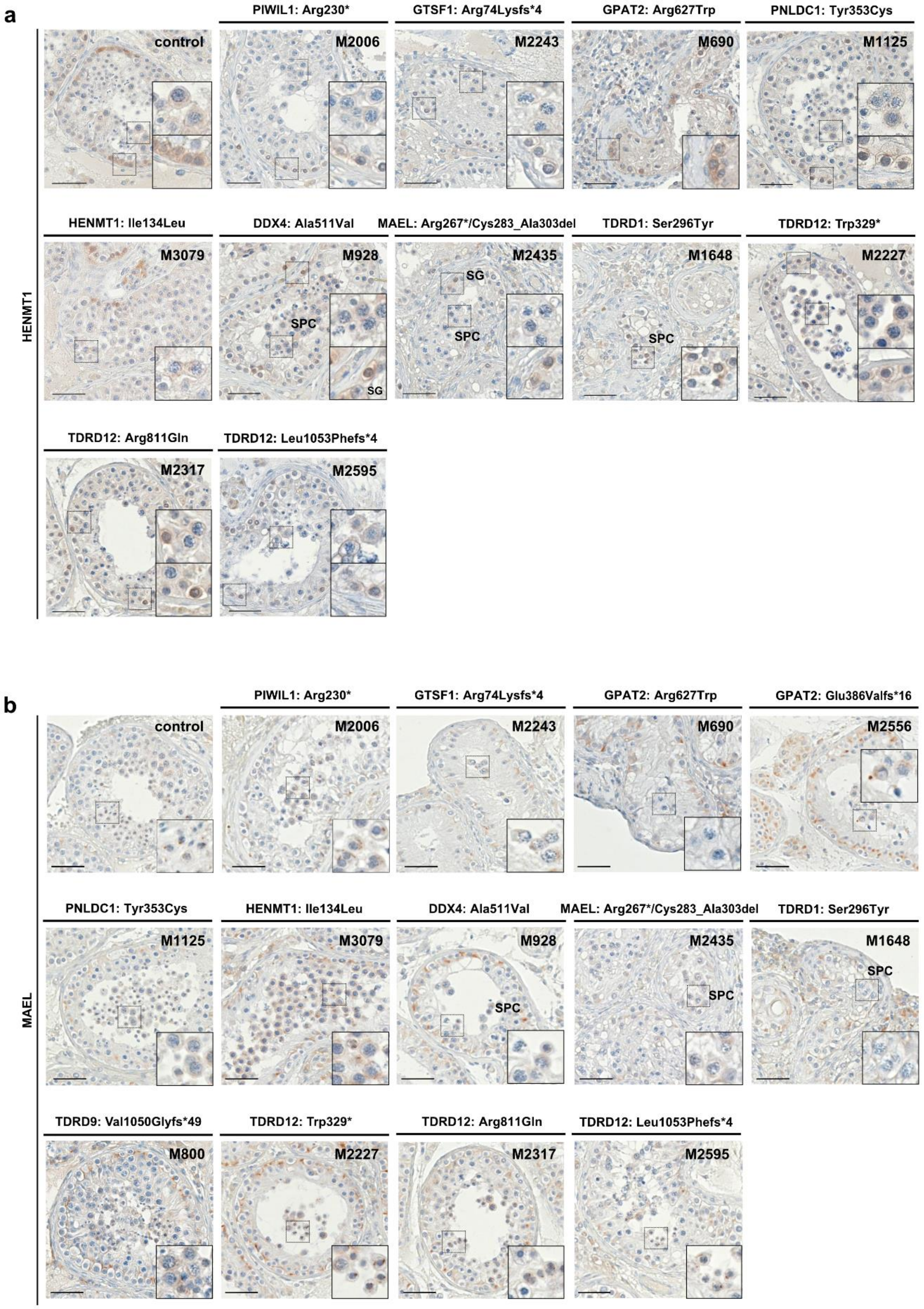


**Supplementary Figure 13. Testicular expression profile of piRNA pathway components HENMT1 and MAEL in control and variant carriers.** a. Immunohistochemical (IHC) staining for HENMT1 in testicular tissue of men with full spermatogenesis (control) and variant carriers. b. IHC staining for MAEL in testicular tissue of men with full spermatogenesis (control) and variant carriers. Representative tubules show the staining pattern observed in independent sections (control: N = 3, proband: N = 2 in case the staining pattern differed from the control) are shown. Scale bar = 50 µm. SG: spermatogonia; SPC: spermatocyte.

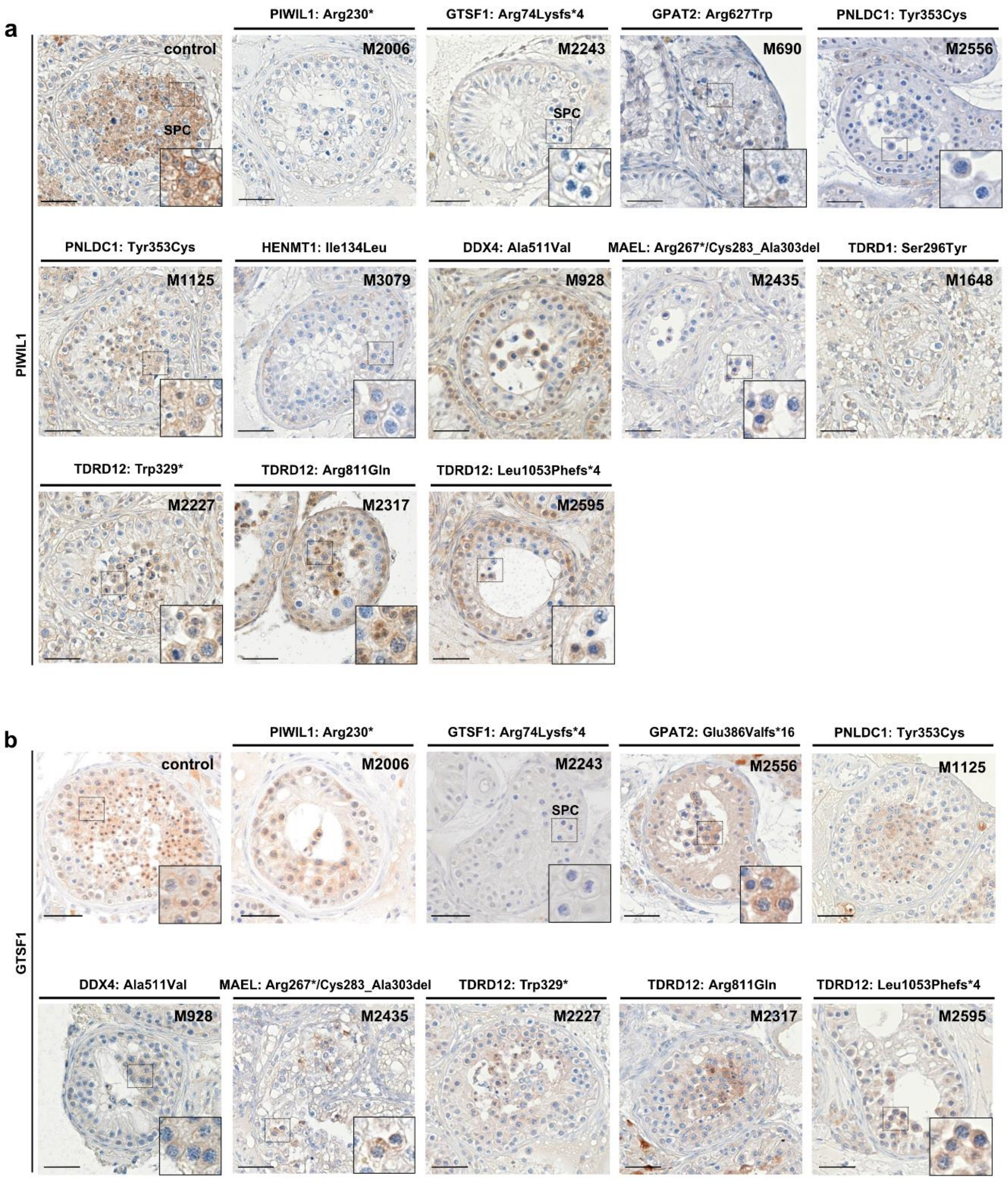


**Supplementary Figure 14. Testicular expression profile of piRNA pathway components PIWIL1 and GTSF1 in controls and variant carriers.** a. Immunohistochemical (IHC) staining of PIWIL1 in testicular tissue of men with full spermatogenesis (control) and variant carriers. b. IHC staining of GTSF1 in testicular tissue of men with full spermatogenesis (control) and variant carriers. Representative tubules show the staining pattern observed in independent sections (control: N = 3, proband: N = 2 in case the staining pattern differed from the control) are shown. Scale bar = 50 µm. SPC: spermatocyte.

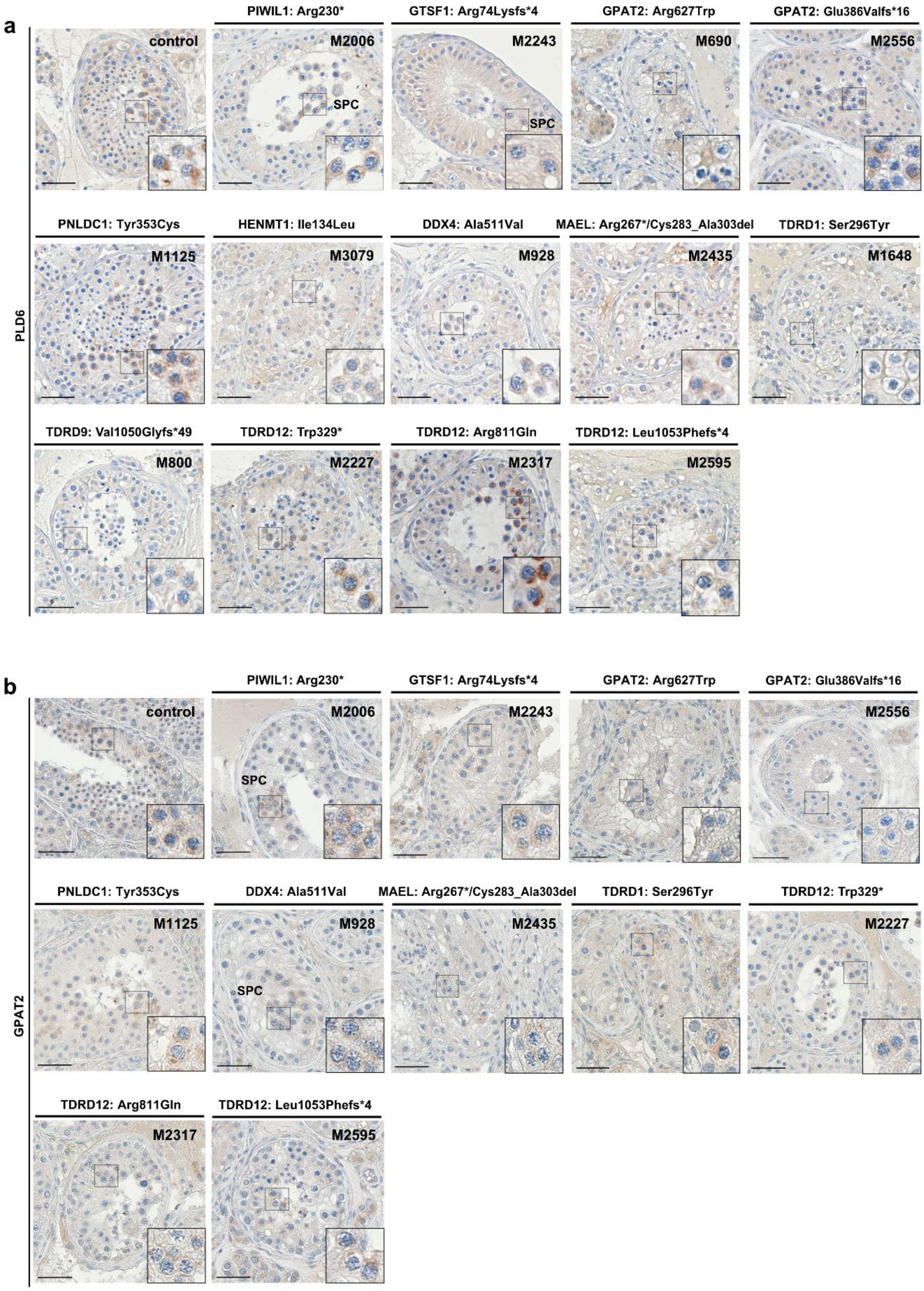


**Supplementary Figure 15. Testicular expression profile of piRNA pathway components PLD6 and GPAT2 in control and variant carriers.** a. Immunohistochemical (IHC) staining of PLD6 in testicular tissue of men with full spermatogenesis (control) and variant carriers. b. IHC staining for GTSF1 in testicular tissue of men with full spermatogenesis (control) and variant carriers. Representative tubules show the staining pattern observed in independent sections (control: N = 3, proband: N = 2 in case the staining pattern differed from the control) are shown. Scale bar = 50 µm. SPC: spermatocyte.

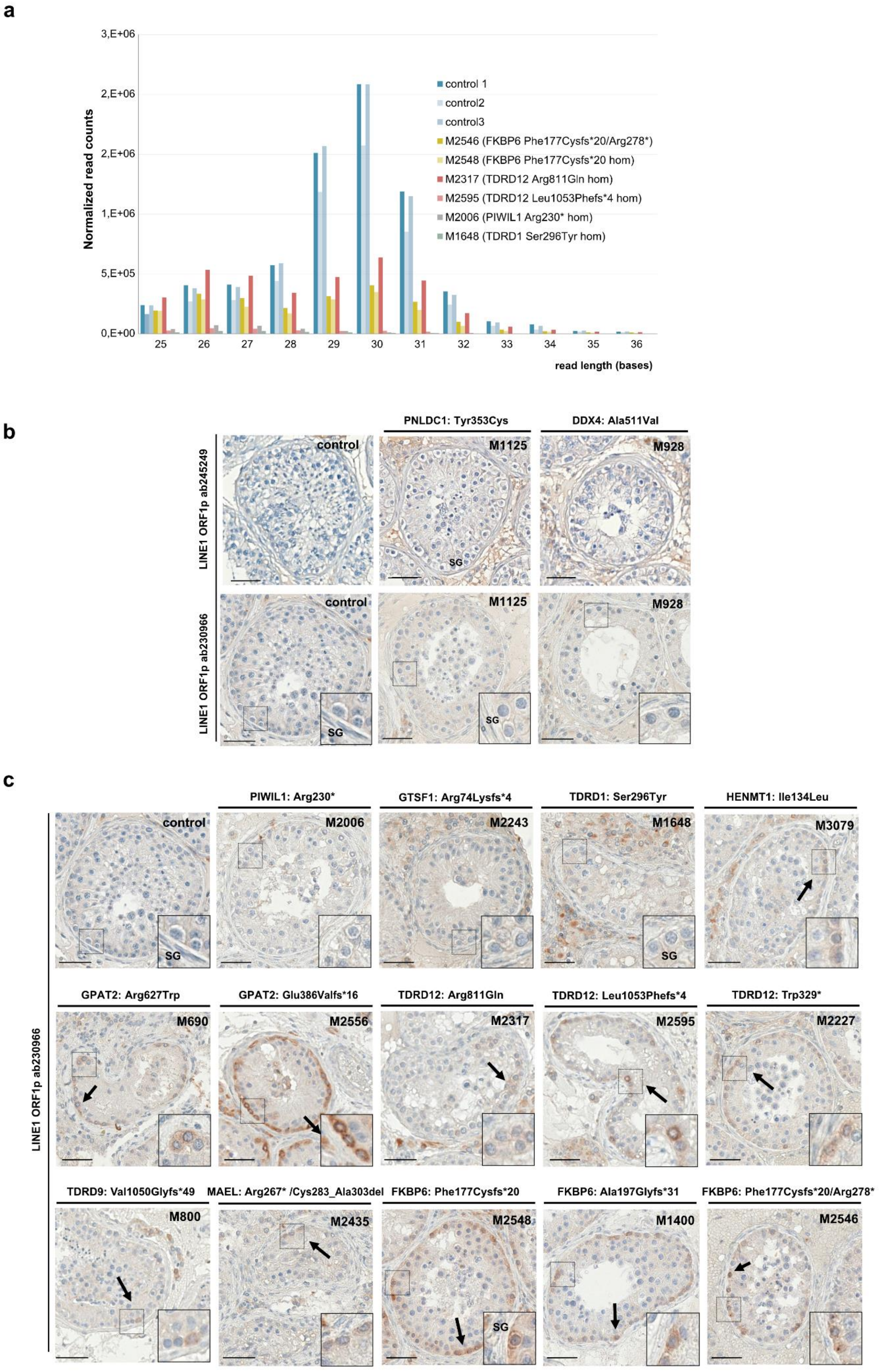
a
3,E+06
2,E+06
2,E+06
1,E+06
5,E+05
0,E+00
Normalized read counts
control 1
control2
control3
M2546 (FKBP6 Phe177Cysfs*20/Arg278*)
M2548 (FKBP6 Phe177Cysfs*20 hom)
M2317 (TDRD12 Arg811Gln hom)
M2595 (TDRD12 Leu1053Phefs*4 hom)
M2006 (PIWIL1 Arg230* hom)
M1648 (TDRD1 Ser296Tyr hom)
25 26 27 28 29 30 31 32 33 34 35 36
read length (bases)
b
PNLDC1: Tyr353Cys
DDX4: Ala511Val
LINE1 ORF1p ab245249
LINE1 ORF1p ab230966
control
M1125
M928
SG
c
PIWIL1: Arg230*
GTSF1: Arg74Lysfs*4
TDRD1: Ser296Tyr
HENMT1: Ile134Leu
control
M2006
M2243
M1648
M3079
GPAT2: Arg627Trp
GPAT2: Glu386Valfs*16
TDRD12: Arg811Gln
TDRD12: Leu1053Phefs*4
TDRD12: Trp329*
M690
M2556
M2317
M2595
M2227
LINE1 ORF1p ab230966
TDRD9: Val1050Glyfs*49
MAEL: Arg267* /Cys283_Ala303del
FKBP6: Phe177Cysfs*20
FKBP6: Ala197Glyfs*31
FKBP6: Phe177Cysfs*20/Arg278*
M800
M2435
M2548
M1400
M2546

**Supplementary Figure 16. Impact of biallelic variants in piRNA biogenesis genes on amount of pachytene piRNA and transposon expression.** a. Amount of pachytene piRNAs with specific lengths in *TDRD12*, *PIWIL1*, and *TDRD1* variant carriers compared to *FKBP6* variant carriers and control tissue with full spermatogenesis. Shapiro-Wilk test was used to estimate the normality of the data. Since Shapiro-Wilk test indicated abnormal data distribution in both control and case groups, two-sided Mann-Whitney U test was used for comparing the expression changes of piRNAs with different length (26-31 nt) between both groups. Source data are provided as a Source Data file. b. Immunohistochemical staining of LINE1 ORF1p in testicular tissue of infertile men with homozygous missense variants in *PNLDC1* and *DDX4*. IHC was performed using monoclonal antibodies ab245249 and ab230966, directed against human LINE1 ORFp1. c. IHC for LINE1 ORF1p in testicular tissue of variant carriers using monoclonal antibody ab230966 confirmed staining pattern results of LINE1 ORF1p ab245249. LINE1 ORF1p was not detected in testicular tissue of controls with full spermatogenesis and PIWIL1, *GTSF1* and *TDRD1* variant carriers. In contrast, both *GPAT2* variant carriers, all three *TDRD12* variant carriers and all three *FKBP6* variant carriers revealed a concordant distinct and specific LINE1 ORF1p staining in spermatogonia. A similar effect was also seen for *MAEL*, *HENMT1*, and *TDRD9* variant carriers. Representative tubules show the staining pattern observed in independent sections (control: N = 3, proband: N = 2) are shown. Scale bar = 50 µm. SG: spermatogonia.

## Supplementary References

1. Deng, W. & Lin, H. miwi, a murine homolog of piwi, encodes a cytoplasmic protein essential for spermatogenesis. *Developmental cell* **2**, 819–30 (2002).

2. Kuramochi-Miyagawa, S. *et al.* Mili, a mammalian member of piwi family gene, is essential for spermatogenesis. *Development (Cambridge, England)* **131**, 839–49 (2004).

3. Carmell, M. A. *et al.* MIWI2 is essential for spermatogenesis and repression of transposons in the mouse male germline. *Developmental cell* **12**, 503–14 (2007).

4. Yoshimura, T. *et al.* Gtsf1/Cue110, a gene encoding a protein with two copies of a CHHC Zn-finger motif, is involved in spermatogenesis and retrotransposon suppression in murine testes. *Developmental biology* **335**, 216–27 (2009).

5. Watanabe, T. *et al.* MITOPLD is a mitochondrial protein essential for nuage formation and piRNA biogenesis in the mouse germline. *Developmental cell* **20**, 364–75 (2011).

6. Shiromoto, Y. *et al.* GPAT2 is required for piRNA biogenesis, transposon silencing, and maintenance of spermatogonia in mice†. *Biology of reproduction* **101**, 248–256 (2019).

7. Zheng, K. *et al.* Mouse MOV10L1 associates with Piwi proteins and is an essential component of the Piwi-interacting RNA (piRNA) pathway. *Proceedings of the National Academy of Sciences of the United States of America* **107**, 11841–6 (2010).

8. Ma, L. *et al.* GASZ is essential for male meiosis and suppression of retrotransposon expression in the male germline. *PLoS genetics* **5**, e1000635 (2009).

9. Nishimura, T. *et al.* PNLDC1, mouse pre-piRNA Trimmer, is required for meiotic and post-meiotic male germ cell development. *EMBO reports* **19**, (2018).

10. Ding, D. *et al.* PNLDC1 is essential for piRNA 3' end trimming and transposon silencing during spermatogenesis in mice. *Nature communications* **8**, 819 (2017).

11. Zhang, Y. *et al.* An essential role for PNLDC1 in piRNA 3' end trimming and male fertility in mice. *Cell research* **27**, 1392–1396 (2017).

12. Lim, S. L. *et al.* HENMT1 and piRNA Stability Are Required for Adult Male Germ Cell Transposon Repression and to Define the Spermatogenic Program in the Mouse. *PLoS genetics* **11**, e1005620 (2015).

13. Xiol, J. *et al.* A role for Fkbp6 and the chaperone machinery in piRNA amplification and transposon silencing. *Molecular cell* **47**, 970–9 (2012).

14. Soper, S. F. C. *et al.* Mouse maelstrom, a component of nuage, is essential for spermatogenesis and transposon repression in meiosis. *Developmental cell* **15**, 285–97 (2008).

15. Kuramochi-Miyagawa, S. *et al.* MVH in piRNA processing and gene silencing of retrotransposons. *Genes & development* **24**, 887–92 (2010).

16. Tanaka, S. S. *et al.* The mouse homolog of Drosophila Vasa is required for the development of male germ cells. *Genes & development* **14**, 841–53 (2000).

17. Reuter, M. *et al.* Loss of the Mili-interacting Tudor domain-containing protein-1 activates transposons and alters the Mili-associated small RNA profile. *Nature structural & molecular biology* **16**, 639–46 (2009).

18. Saxe, J. P., Chen, M., Zhao, H. & Lin, H. Tdrkh is essential for spermatogenesis and

participates in primary piRNA biogenesis in the germline. *The EMBO journal* **32**, 1869–85 (2013).

19. Pan, J. *et al.* RNF17, a component of the mammalian germ cell nuage, is essential for spermiogenesis. *Development (Cambridge, England)* **132**, 4029–39 (2005).

20. Ding, D. *et al.* TDRD5 binds piRNA precursors and selectively enhances pachytene piRNA processing in mice. *Nature communications* **9**, 127 (2018).

21. Yabuta, Y. *et al.* TDRD5 is required for retrotransposon silencing, chromatoid body assembly, and spermiogenesis in mice. *The Journal of cell biology* **192**, 781–95 (2011).

22. Shoji, M. *et al.* The TDRD9-MIWI2 complex is essential for piRNA-mediated retrotransposon silencing in the mouse male germline. *Developmental cell* **17**, 775–87 (2009).

23. Pandey, R. R. *et al.* Tudor domain containing 12 (TDRD12) is essential for secondary PIWI interacting RNA biogenesis in mice. *Proceedings of the National Academy of Sciences of the United States of America* **110**, 16492–7 (2013).

24. Bolcun-Filas, E. *et al.* A-MYB (MYBL1) transcription factor is a master regulator of male meiosis. *Development (Cambridge, England)* **138**, 3319–30 (2011).

25. Dong, J. *et al.* UHRF1 suppresses retrotransposons and cooperates with PRMT5 and PIWI proteins in male germ cells. *Nature communications* **10**, 4705 (2019).

26. Ichiyanagi, T. *et al.* HSP90α plays an important role in piRNA biogenesis and retrotransposon repression in mouse. *Nucleic acids research* **42**, 11903–11 (2014).

27. Zhou, L. *et al.* BTBD18 Regulates a Subset of piRNA-Generating Loci through Transcription Elongation in Mice. *Developmental cell* **40**, 453-466.e5 (2017).

28. Wyrwoll, M. J. *et al.* The piRNA-pathway factor FKBP6 is essential for spermatogenesis but dispensable for control of meiotic LINE-1 expression in humans. *American journal of human genetics* **109**, 1850–1866 (2022).

29. Nagirnaja, L. *et al.* Diverse monogenic subforms of human spermatogenic failure. *Nature communications* **13**, 7953 (2022).

30. Huang, H. *et al.* piRNA-associated germline nuage formation and spermatogenesis require MitoPLD profusogenic mitochondrial-surface lipid signaling. *Developmental cell* **20**, 376–87 (2011).

31. Li, Y., Zhang, Y. & Liu, M. Knockout Gene-Based Evidence for PIWI-Interacting RNA Pathway in Mammals. *Frontiers in cell and developmental biology* **9**, 681188 (2021).

32. Arif, A. *et al.* GTSF1 accelerates target RNA cleavage by PIWI-clade Argonaute proteins. *Nature* **608**, 618–625 (2022).

33. Ozata, D. M., Gainetdinov, I., Zoch, A., O'Carroll, D. & Zamore, P. D. PIWI-interacting RNAs: small RNAs with big functions. *Nature reviews. Genetics* **20**, 89–108 (2019).

34. Oud, M. S. *et al.* A de novo paradigm for male infertility. *Nature Communications* (2022) doi:10.1038/s41467-021-27132-8.
