# Supplementary material for: Inherited defects of piRNA biogenesis cause transposon de-repression, impaired spermatogenesis, and human male infertility
Source: Nat Commun. 2024 Aug 9;15:6637. doi: 10.1038/s41467-024-50930-9 (PMC11316121; doi:10.1038/s41467-024-50930-9)
Supplement: Supplementary file 3 — Description of additional supplementary files [file 41467_2024_50930_MOESM3_ESM.pdf]

Description of Additional Supplementary Files

File name: Supplementary Data 1

Description: Characteristics of identified high-impact variants in genes of the piRNA pathway and genetic constraints of affected genes

File name: Supplementary Data 2

Description: .pdb file of protein structure for PNLDC1 (NM_001271862.2)

File name: Supplementary Data 3

Description: .pdb file of protein structure for GPAT2 NM_001321526.1

File name: Supplementary Data 4

Description: Accession codes and links of Data Availability statement
