# Supplementary material for: Inherited defects of piRNA biogenesis cause transposon de-repression, impaired spermatogenesis, and human male infertility
Source: Nat Commun. 2024 Aug 9;15:6637. doi: 10.1038/s41467-024-50930-9 (PMC11316121; doi:10.1038/s41467-024-50930-9)
Supplement: Supplementary file 8 — Reporting Summary [file 41467_2024_50930_MOESM8_ESM.pdf]

nature portfolio

Corresponding author(s): Frank Tüttelmann

Last updated by author(s): Jul 9, 2024

Reporting Summary

Nature Portfolio wishes to improve the reproducibility of the work that we publish. This form provides structure for consistency and transparency in reporting. For further information on Nature Portfolio policies, see our Editorial Policies and the Editorial Policy Checklist.

Statistics

For all statistical analyses, confirm that the following items are present in the figure legend, table legend, main text, or Methods section.

n/a	Confirmed	
☐	☒	The exact sample size (*n*) for each experimental group/condition, given as a discrete number and unit of measurement
☒	☐	A statement on whether measurements were taken from distinct samples or whether the same sample was measured repeatedly
☐	☒	The statistical test(s) used AND whether they are one- or two-sided<br>*Only common tests should be described solely by name; describe more complex techniques in the Methods section.*
☒	☐	A description of all covariates tested
☐	☒	A description of any assumptions or corrections, such as tests of normality and adjustment for multiple comparisons
☒	☐	A full description of the statistical parameters including central tendency (e.g. means) or other basic estimates (e.g. regression coefficient) AND variation (e.g. standard deviation) or associated estimates of uncertainty (e.g. confidence intervals)
☐	☒	For null hypothesis testing, the test statistic (e.g. *F*, *t*, *r*) with confidence intervals, effect sizes, degrees of freedom and *P* value noted<br>*Give P values as exact values whenever suitable.*
☒	☐	For Bayesian analysis, information on the choice of priors and Markov chain Monte Carlo settings
☒	☐	For hierarchical and complex designs, identification of the appropriate level for tests and full reporting of outcomes
☒	☐	Estimates of effect sizes (e.g. Cohen's *d*, Pearson's *r*), indicating how they were calculated

Our web collection on statistics for biologists contains articles on many of the points above.

Software and code

Policy information about availability of computer code

Data collection	no specific software was used for data collection
Data analysis	exome/genom data analysis: trimming of remaining adapter sequences: Cutadapt v1.15; Alignment: BWA Mem v0.7.17 or llumina Dragen Bio-IT platform v4.2. Variant calling: GATK toolkit v3.8 or or llumina Dragen Bio-IT platform v4.2. Variant annotation: Ensembl Variant Effect Predictor<br>small RNA seq: trimmed reads were aligned to reference genome (GRCh37) with Bowtie (v.#1.0.1) and known small non-coding RNAs, other than piRNAs, were removed from the dataset using DASHRv2 (v.#v2)

For manuscripts utilizing custom algorithms or software that are central to the research but not yet described in published literature, software must be made available to editors and reviewers. We strongly encourage code deposition in a community repository (e.g. GitHub). See the Nature Portfolio guidelines for submitting code & software for further information.

Data

Policy information about availability of data

All manuscripts must include a data availability statement. This statement should provide the following information, where applicable:

- Accession codes, unique identifiers, or web links for publicly available datasets
- A description of any restrictions on data availability
- For clinical datasets or third party data, please ensure that the statement adheres to our policy

All links for each individual accession code are provided in Supplementary Data 4. The new genetic variants indicated in this study have been submitted to ClinVar under the accession numbers SCV004698009 - SCV004698043. ClinVar accession codes for already published genetic variants in FKBP6 are SCV002507290, SCV002507292, and SCV002507294. Submission of human exome/genome sequencing data from the MERGE cohort, the Strasbourg cohort, and the Barcelona cohort to a repository is not covered by the probands informed consent. These data will be available from the corresponding author upon request. Sequencing data from the Nijmegen cohort and piRNA-seq data (.fastq files) have been deposited in the European Genome-phenome Archive (EGA) under the accession codes EGAS00001005417 and EGAS50000000397. These will be made available upon request for academic use and within the limitations of the provided informed consent by applying for access through the EGA's online form. Every request will be reviewed within four weeks by the respective Data Access Committee and the researcher will need to sign a data access agreement after approval.. Accession codes for AlphaFold2 structures are AF-Q9NQI0-F1 for DDX4, AF-Q8WW33-F1 for GTSF1, AF-Q5T8I9-F1 for HENMT1, AF-Q9BXT6-F1 for MOV10L1, AF-Q8TC59-F1 for PIWIL2, AF-Q9BXT4-F1 for TDRD1, AF-Q8NDG6-F1 for TDRD9, AF-Q587J7-F1 for TDRD12. For GPAT2 (NM_001321526.1), the .pdb file is provided as Supplementary Data 2. For PNLDC1 (NM_001271862.2), the .pdb file is provided as Supplementary Data 3. Source data are provided with this paper.

Research involving human participants, their data, or biological material

Policy information about studies with human participants or human data. See also policy information about sex, gender (identity/presentation), and sexual orientation and race, ethnicity and racism.

Reporting on sex and gender	findings presented in this manuscript apply only to male sex
Reporting on race, ethnicity, or other socially relevant groupings	no analysis was made on the study cohort in respect to race
Population characteristics	most of the patients included in this study are of European origin
Recruitment	All study cohorts are based on a prospective recruitment of men who attended andrological examination because of infertility. Most patients (>90%) were recruited at the Centre of Reproductive Medicine and Andrology (CeRA), University Hospital Münster. In addition patients were recruited at the Clinic for Urology, Paediatric Urology and Andrology, Justus Liebig University Gießen, the Andrology Department, Fundació Puigvert, Universitat Autònoma de Barcelona, the Department of Obstetrics and Gynecology, Radboud University Medical Center, Nijmegen and the Department of Andrology Novafertil IVF Center, Konya
Ethics oversight	All persons gave written consent compliant with local requirements. The study protocol was approved by the local ethics committees: MERGE Münster (2010-578-f-S) and Giessen (26/11); Strasbourg (CPP 09/40—WAC-2008-438 1W DC-2009-I 002), and Yeni Yüzyıl University, Scientific, social and noninterventional health sciences research ethics committee, Istanbul, Turkey (approval no: 2019/08); Barcelona: (2014/04c); Newcastle: (Newcastle:REC ref. 18/NE/0089), Nijmegen: (NL50495.091.14 version 5.0).

Note that full information on the approval of the study protocol must also be provided in the manuscript.

Field-specific reporting

Please select the one below that is the best fit for your research. If you are not sure, read the appropriate sections before making your selection.

☒ Life sciences ☐ Behavioural & social sciences ☐ Ecological, evolutionary & environmental sciences

For a reference copy of the document with all sections, see nature.com/documents/nr-reporting-summary-flat.pdf

Life sciences study design

All studies must disclose on these points even when the disclosure is negative.

Sample size	Sample size refers to infertile men of the resepective cohort included in genetic analysis. For MERGE most of this cohort are azoospermic (N = 1,448) or have severely reduced sperm counts: N = 454 with cryptozoospermia (sperm only identified after centrifugation of the ejaculate); N = 158 with extreme oligozoospermia (sperm count <2 million); N = 67 with severe oligozoospermic (sperm count <10 million).
Data exclusions	no data were excluded
Replication	all genetic variants described in this manuscript were identified by NGS and confirmed by Sanger sequencing; IIHC results in patients testicular sections were confirmed differed from the staining pattern seen in control sections with full spermatogenesis these results were confirmed in

at least one additional section. IHC results that differed from the staining pattern repeatedly seen in testicular tissue with full spermatogenesis were confirmed. If the stining pattern observed in a patient was similar to the staining pattern seen in control testicular tissue the result was not confirmed as patient testicular biopsy material was rare. piRNAseq analysis was not confirmed as patient material was unsufficient fo repeat RNA extraction.

Randomization: not applicable

Blinding: For all experiments, the investigators were not blinded to group allocation during data collection and/or analysis but assessment of clinical data and genetic analysis was performed by independent researchers.

Reporting for specific materials, systems and methods

We require information from authors about some types of materials, experimental systems and methods used in many studies. Here, indicate whether each material, system or method listed is relevant to your study. If you are not sure if a list item applies to your research, read the appropriate section before selecting a response.

Materials & experimental systems

n/a	Involved in the study	
☐	☒	Antibodies
☐	☒	Eukaryotic cell lines
☒	☐	Palaeontology and archaeology
☒	☐	Animals and other organisms
☒	☐	Clinical data
☒	☐	Dual use research of concern
☒	☐	Plants

Methods

n/a	Involved in the study	
☒	☐	ChIP-seq
☒	☐	Flow cytometry
☒	☐	MRI-based neuroimaging

Antibodies

Antibodies used:

CREM: Sigma Aldrich, #HPA001818 (LOT: A71668), rabbit, polyclonal, dilution 1:2000 in 5% BSA/TBS
γH2AX: Merck, #05-636 (LOT: 3866252), mouse, monoclonal, dilution 1:50 in TBS + 0,1% Tween
PIWIL1: Invitrogen, #MA5-41250 (LOT: XB3515977A), rabbit, polyclonal, dilution 1:200 in 25% goat serum in 0,5% BSA/TBS
GTSF1: ATLAS antibodies, #HPA038876 (LOT: 17490), rabbit, polyclonal, , dilution 1:50 in 0,5% BSA/TBS
TDRD1: Antibodies.com, #A54784 (LOT: 2089.PB1.AP), rabbit, polyclonal, dilution 1:100 in 5% BSA/TBS
HENMT1: Invitrogen, #PA5-55866 (LOT: A118330), rabbit, polyclonal, dilution 1:150 in 25% goat serum in 5% BSA/TBS
GPAT2: Sigma Aldrich, #HPA036841 (LOT: 21653), rabbit, polyclonal, dilution 1:50 in 25% goat serum in 5% BSA/TBS
DDX4: Abcam, #ab13840 (LOT: GR3274948-1), rabbit, polyclonal, dilution 1:2000 in 5% BSA/TBS
MAEL: Novus Biologicals, #NBP2-69070 (LOT: R113395), rabbit, polyclonal, dilution 1:300 in 5% BSA/TBS
LINE-1 ORF1p: Abcam, #ab245249 (LOT: GR3309493-3), rabbit, monoclonal, , dilution1:100 in 25% goat serum in 5% BSA/TBS
LINE-1 ORF1p: Abcam, #ab ab230966 (LOT:1020682-6), rabbit, monoclonal, , dilution 1:200 -1:500 in 25% goat serum in 5% BSA/TBS
PLD6: Novus Biologicals, #NBP2-13771 (LOT: A104980), rabbit, polyclonal, dilution 1:100 in 25% goat serum in 5% BSA/TBS
GAPDG: Cell signalling, #5174 (LOT:8), rabbit, monoclonal 1:1500 in 5% milk powder in TBST
HA-tag: Roche Sigma, #11867423 (LOT: 65506600), rat monoclonal, 1:2000 in 5% milk powder in TBST

Validation:

CREM: Antibody Enhanced-Validation including Orthogonal Validation Using RNA-Seq
γH2AX: PMID: 34755185; PMID: 26072710
PIWIL1: https://www.thermofisher.com/antibody/product/PIWIL1-Antibody-clone-JU35-12-Recombinant-Monoclonal/MA5-41250; applications: WB, IHC (paraffin); staining in human testis corresponds to scRNAseq data; staining is absent in testicular tissue of patient with homozygous LoF variant in PIWIL1 (this study)
GTSF1: validated for IHC (Human protein atlas); staining pattern corresponds to scRNAseq data; staining is absent in testicular tissue of patient with homozygous LoF variant in GTSF1 (this study)
TDRD1: https://www.antibodies.com/de/tdrd1-antibody-a54784; applications: IHC, ELISA, WB; staining pattern corresponds to scRNAseq data
HENMT1: https://www.thermofisher.com/antibody/product/HENMT1-Antibody-Polyclonal/PA5-55866; applications: WB, IHC; staining pattern corresponds to scRNAseq data
GPAT2: https://www.sigmaaldrich.com/DE/de/product/sigma/hpa036841; applications: IHC, staining pattern corresponds to scRNAseq data; staining is absent in testicular tissue of patients with homozygous variant in GPAT2
DDX4: https://www.abcam.com/products/primary-antibodies/ddx4--mvh-antibody-ab13840.html; applications: WB, ICC/IF, IHC; PMID: 18088417
MAEL: https://www.novusbio.com/products/mael-antibody_nbp2-69070; applications: IHC, staining pattern corresponds to scRNAseq data, staining is absent in testicular tissue of patient with biallelic LoF variant (this study)
LINE-1 ORF1p: https://www.abcam.com/products/primary-antibodies/line-1-orf1p-antibody-epr22227-6-ab245249; applications: Flow Cyt, WB, IHC-P, ICC/IF, IP
LINE-1 ORF1p ab230966: https://www.abcam.com/en-de/products/primary-antibodies/line-1-orf1p-antibody-epr22227-54-ab230966
applications: IP, WB, ICC/IF, Flow Cyt (Intra), IHC-P
PLD6: https://www.novusbio.com/PDFs/NBP2-13771.pdf; applications: IHC

GAPDH: https://www.cellsignal.com/products/primary-antibodies/gapdh-d16h11-xp-rabbit-mab/5174; applications: WB, IHC, IF
HA-tag: https://www.sigmaaldrich.com/DE/de/product/roche/roaha?gclid=EAIaIQobChMIq4Oq67aXhwMVQQcGAB28FQQMEAAYASAAEgKum_D_BwE; applications: WB

Eukaryotic cell lines

Policy information about cell lines and Sex and Gender in Research

Cell line source(s)	HEK293T Lenti-X, Clontech Laboratories; catalog number: 632180
Authentication	the cells were not authenticated
Mycoplasma contamination	cell lines were negative for mycoplasm contamination
Commonly misidentified lines (See ICLAC register)	no commonly misidentified cell lines were used

Plants

Seed stocks	not applicable
Novel plant genotypes	not applicable
Authentication	not applicable
